# The Ethylene Precursor ACC Affects Early Vegetative Development Independently of Ethylene Signaling

**DOI:** 10.3389/fpls.2019.01591

**Published:** 2019-12-06

**Authors:** Lisa Vanderstraeten, Thomas Depaepe, Sophie Bertrand, Dominique Van Der Straeten

**Affiliations:** Laboratory of Functional Plant Biology, Department of Biology, Ghent University, Ghent, Belgium

**Keywords:** 1-aminocyclopropane-1-carboxylic acid, ethylene signaling, root growth, rosette growth, triple response, vegetative growth

## Abstract

The plant hormone ethylene plays a pivotal role in virtually every aspect of plant development, including vegetative growth, fruit ripening, senescence, and abscission. Moreover, it acts as a primary defense signal during plant stress. Being a volatile, its immediate biosynthetic precursor, 1-aminocyclopropane-1-carboxylic acid, ACC, is generally employed as a tool to provoke ethylene responses. However, several reports propose a role for ACC in parallel or independently of ethylene signaling. In this study, pharmacological experiments with ethylene biosynthesis and signaling inhibitors, 2-aminoisobutyric acid and 1-methylcyclopropene, as well as mutant analyses demonstrate ACC-specific but ethylene-independent growth responses in both dark- and light-grown *Arabidopsis* seedlings. Detection of ethylene emanation in ethylene-deficient seedlings by means of laser-based photoacoustic spectroscopy further supports a signaling role for ACC. In view of these results, future studies employing ACC as a proxy for ethylene should consider ethylene-independent effects as well. The use of multiple knockout lines of ethylene biosynthesis genes will aid in the elucidation of the physiological roles of ACC as a signaling molecule in addition to its function as an ethylene precursor.

## Introduction

The gaseous plant hormone ethylene has been shown to regulate a myriad of physiological and developmental processes including germination, root growth and root hair formation, leaf expansion, leaf and flower senescence, abscission, fruit ripening, nodulation, and the response to numerous stresses (Burg and Burg, 1962; Abeles et al., 1992; Vandenbussche et al., 2012). Ethylene is formed from the amino acid methionine in three subsequent steps with S-adenosylmethionine (SAM) and 1-aminocyclopropane-1-carboxylic acid (ACC) as intermediates. The rate-limiting step in the biosynthesis of ethylene is the conversion of SAM to ACC, catalyzed by the enzyme ACC synthase (ACS) (Boller et al., 1979; Yang and Hoffman, 1984). In *Arabidopsis*, ACS proteins are encoded by a multigene family, eight of which are functional ACC synthases. The transcription of *ACS* genes is highly regulated during plant development and in response to a wide variety of developmental, hormonal, and environmental stimuli (Liang et al., 1992; Van Der Straeten et al., 1992; Tsuchisaka and Theologis, 2004). The final step of ethylene biosynthesis, the oxidation of ACC to ethylene, is catalyzed by the enzyme ACC oxidase (ACO) (Ververidis and John, 1991). In *Arabidopsis*, the *ACO* gene family consists of five members that are also differentially regulated (Barry et al., 1996; Nakatsuka et al., 1998). Although ACS is the major rate-limiting enzyme in ethylene biosynthesis, under certain conditions, for example, during fruit ripening, ACO can also become rate-limiting (Barry et al., 1996; Van De Poel et al., 2012). Moreover, the levels of ACC are not only regulated at the level of ACS and ACO activity, but are also dependent on conjugation and deamination of ACC (Amrhein et al., 1981; Martin et al., 1995; Glick et al., 1998; Mcdonnell et al., 2009).

As the immediate and water-soluble precursor of ethylene, the main role of ACC is to act as a mobile signal for short- and long-distance communication within the plant. Transport of ACC throughout the plant has been observed in numerous cases (Bradford and Yang, 1980; Lurssen, 1981; Zarembinski and Theologis, 1993; Morris and Larcombe, 1995; Jackson, 2002; Almeida et al., 2003; Jackson, 2008; Vanderstraeten and Van Der Straeten, 2017). Recently, the amino acid transporter LYSINE HISTIDINE TRANSPORTER1 (LHT1) has been demonstrated to transport ACC in etiolated *Arabidopsis* seedlings (Shin et al., 2015). While it is clear that a major role of ACC is to act as the precursor of ethylene, several studies suggest that ACC itself can act as a signal independent of its oxidation to ethylene. Exogenous ACC is widely applied as a tool to study ethylene responses in plants. Both the triple response phenotype in etiolated seedlings and the reduced rosette size in light-grown plantlets, typical ethylene-related phenotypes, are triggered by ACC as well (Guzman and Ecker, 1990; Van Der Straeten et al., 1993; Roman et al., 1995; Smalle et al., 1997). The comparison of null mutations in key ethylene signaling components and the octuple *ACS* (*acs8x*) ethylene biosynthesis mutant revealed that not ethylene but ACC is crucial for viability, since only the latter resulted in embryo lethality (Tsuchisaka et al., 2009). Moreover, Xu et al. (2008) suggest that ACC might act as a signaling molecule to regulate cell expansion in the FEI/SOS5 pathway. Investigating the *fei1fei2* mutant they found that the cell expansion phenotypes in roots could be reversed by blocking ethylene biosynthesis [using AOA (2-aminooxyacetic acid, an ACS inhibitor) or AIB (2-aminoisobutyric acid, an ACO inhibitor)] but could not be reversed by chemical [using 1-MCP (1-methylcyclopropene) or silver thiosulfate] or genetic (using *etr1* or *ein2* ethylene insensitive mutants) disruption of ethylene perception. A couple of years later, Tsang et al. (2011) observed that the short-term response to cell wall damage or PAMPs resulting in rapid reduction of primary root elongation depends on the biosynthesis of ACC but is independent of the perception of ethylene. They were able to show that AIB is capable of fully restoring the LEH (length of the first epidermal cell with a visible root hair bulge) in isoxaben-treated (inhibitor of cellulose biosynthesis) roots but did not affect the ACC response. Recently, a signaling role for ACC in stomatal development has been demonstrated (Yin et al., 2019). The symmetric division of the guard mother cell (GMC) into two guard cells represents the last step in stomatal development, a process depending on ACC. Pharmacological manipulation of ACC levels showed that ACC acts as a positive regulator in GMC division. Reduced levels of ACC, in the multiple *acs* knockout lines increased the occurrence of single guard cells (SGC). This phenotype could be rescued by addition of ACC but not by treating SGCs with the ethylene-releasing chemical ethephon. Altogether, these reports demand for a reassessment of the physiological role of ACC as a signaling molecule. In this study, the ethylene-independent signaling role of ACC has been investigated during early vegetative growth. Specifically, ACC negatively affected both rosette development and hypocotyl growth, and inhibited primary root elongation independently of ethylene perception. However, similar to ethylene dose-dependent growth inhibitory effects, roots were more sensitive to ACC compared to shoots.

## Materials and Methods

### Plant Material and Growth Conditions

*Arabidopsis thaliana* (L.) Heynh. Columbia (Col-0) was used as wild-type (WT) in this study. Col-0, *ein2-1* (Roman et al., 1995) and *acs1-1acs2-1acs4-1acs5-2acs6-1acs7-1acs9-1acs11* (*acs8x*; Tsuchisaka et al., 2009), both in Col-0 background, were obtained from the Nottingham Arabidopsis Stock Center (NASC; arabidopsis.info/). Seeds were surface-sterilized for 12 min in a bleach solution containing 5% NaOCl and subsequently washed at least 3 times with sterile distilled water. Seeds were plated on half-strength Murashige and Skoog (1/2 × MS) medium containing 0% (**Figure S1**) or 1% sucrose (all other assays) and 0.8% agar and supplemented with the indicated chemicals. 1-aminocyclopropane-1-carboxylic acid (ACC) and 2-aminoisobutyric acid (AIB) were purchased from Sigma-Aldrich and stock solutions were prepared in distilled water. After a stratification of 3 days at 4°C, plates were transferred to a tissue culture chamber (21°C; 16/8-h photoperiod; 70 µmol s^−1^ m^−2^) for the desired time. For assays with dark-grown seedlings, seeds were exposed to light for 6 h after stratification to induce germination and were subsequently grown for 4 days in complete darkness (21°C).

### Gas Treatments

#### 1-MCP

At the start of the experiment, plates were transferred to a dedicated gassing chamber (Van Cleven, Belgium) containing both a treatment and a control cell. Plants were treated with 1-methylcyclopropene (1-MCP) in the treatment cell for 20 h, followed by 4 h of flushing with 1-MCP-free air. The required amount of 1-MCP (EthylBloc) was dissolved in a buffer containing 0.9% KOH and 0.9% NaOH in a 200-ml beaker, which was immediately transferred to the chamber, to give a final concentration of 50, 100, or 250 ppm inside the treatment cell. In parallel, control plants were placed in the control cell and flushed continuously with 1-MCP-free air. The treatment was repeated on a daily basis until the end of the experiment.

#### Ethylene

Ethylene treatment on 2-week-old plants (**Figure S3**) was carried out in the gassing chambers described above. For combined treatments, plants were first treated with ethylene (Air Liquide, Belgium) for 4 h at a final concentration of 100 ppm, and subsequently treated with 1-MCP for 20 h. Both treatments were repeated on a daily basis until the end of the experiment.

### Phenotypic Analysis

Plants were photographed with a Canon EOS 550D camera (Canon, Tokyo, Japan)) after 14 days of growth on horizontally standing plates. To evaluate effects on shoot growth, rosette area was measured with Rosette Tracker (De Vylder et al., 2012), an open-source plug-in in ImageJ (National Institutes of Health). In addition, root length was measured in ImageJ. The triple response assay was carried out, as described previously (Hu et al., 2017), to evaluate the growth of etiolated seedlings. The length of 4-day-old hypocotyls and roots were measured in ImageJ. Average values were obtained from three independent replicates.

### Measurement of Ethylene Emanation

Ethylene emanation was monitored essentially as described in Van de Poel and Van Der Straeten (2017). For the detection of ethylene levels emitted by etiolated seedlings, approximately 30 seeds were sown in 10-ml chromatography vials (Chromacol, VWR) containing 5 ml ½ × MS medium (four independent biological repeats), transferred to a sterile box and grown in darkness at 21°C. Average values were obtained from four independent replicates. For the detection of ethylene levels in 14-day-old plants, three seeds were sown in 10-ml vials, containing 8 ml ½ × MS medium, and grown in a tissue culture chamber (21°C; 16/8-h photoperiod; 70 µmol s^−1^ m^−2^). Average values were obtained from eight independent replicates 24 h before the start of the measurement, vials were sealed off with a rubber septum and a snap-cap (Chromacol, VWR) to allow ethylene accumulation. Ethylene levels were analyzed by means of laser-based photoacoustic spectroscopy (ETD-300, Sensor Sense, The Netherlands).

### Ethylene Complementation Assay

To monitor residual ethylene biosynthesis in the presence of ACC and the ACO blocker AIB, ethylene emission was examined, as described above, in etiolated Col-0 seedlings on a daily basis. The effect of this concentration of ethylene was assessed as follows. Col-0 seeds were sown in 10-ml chromatography vials on media containing 0, 10, or 50 µM ACC in the absence or presence of 2 mM AIB. After germination, ethylene was injected with a gas-tight syringe (Hamilton) to a final concentration equivalent to the levels measured, over a 24-h period from day 3 to day 4, upon treating with 10 [= ETH(10)] or 50 µM ACC [= ETH(50)] in the presence of 2 mM AIB. The final concentrations were 116 and 585 ppb, respectively. Seedlings were allowed to grow for 3 days in complete darkness, after which the phenotypic effects were evaluated at the level of hypocotyl and root growth. Seedlings were grown in the same vials on media containing ACC and/or AIB with ethylene-free air as a control. Average values were obtained from four independent replicates.

### Statistical Analysis

All statistical analyses were carried out in the free software environment for statistical computing and graphics R 3.2.3 (R Foundation for Statistical Computing, Vienna, Austria, www.R-project.org). Data are presented as means, error bars are standard deviations. Statistical analysis comparing two means was performed using the Wilcoxon rank sum test/T-test (P < 0.01). Statistical analysis comparing multiple means was performed using One-Way ANOVA/Kruskal-Wallis (one independent variable) or Scheirer-Ray-Hare (two independent variables) tests (*P* < 0.01) followed by *post hoc* Tukey’s HSD/Dunn tests (*P* < 0.01) with Benjamini and Hochberg correction for multiple pairwise comparisons. In addition, effect sizes for multifactorial analyses are presented and interpreted with partial η² according to Richardson (2011). Small, medium, and large effects correspond with effect sizes of 0.01, 0.06, and 0.15, respectively. Partial η² is calculated as the sum of squares (SS) divided by the sum of SS and SS of the residuals. Effect size for comparisons between two groups is presented as r (Rosenthal, 1994). R was calculated as $Z/\sqrt{N}$ (Wilcoxon rank sum tests, Tukey’s HSD and Dunn tests) or as √(t^2/(t^2+df)) (T-tests). Small, medium, and large effects correspond with effect sizes of 0.1, 0.3, and >0.5, respectively. Relevant output of effect sizes can be found in **Table S1**.

## Results

### Dose-Dependent Effects of ACC on Shoot and Root Development Upon Ethylene Insensitivity

**Figure 1:**
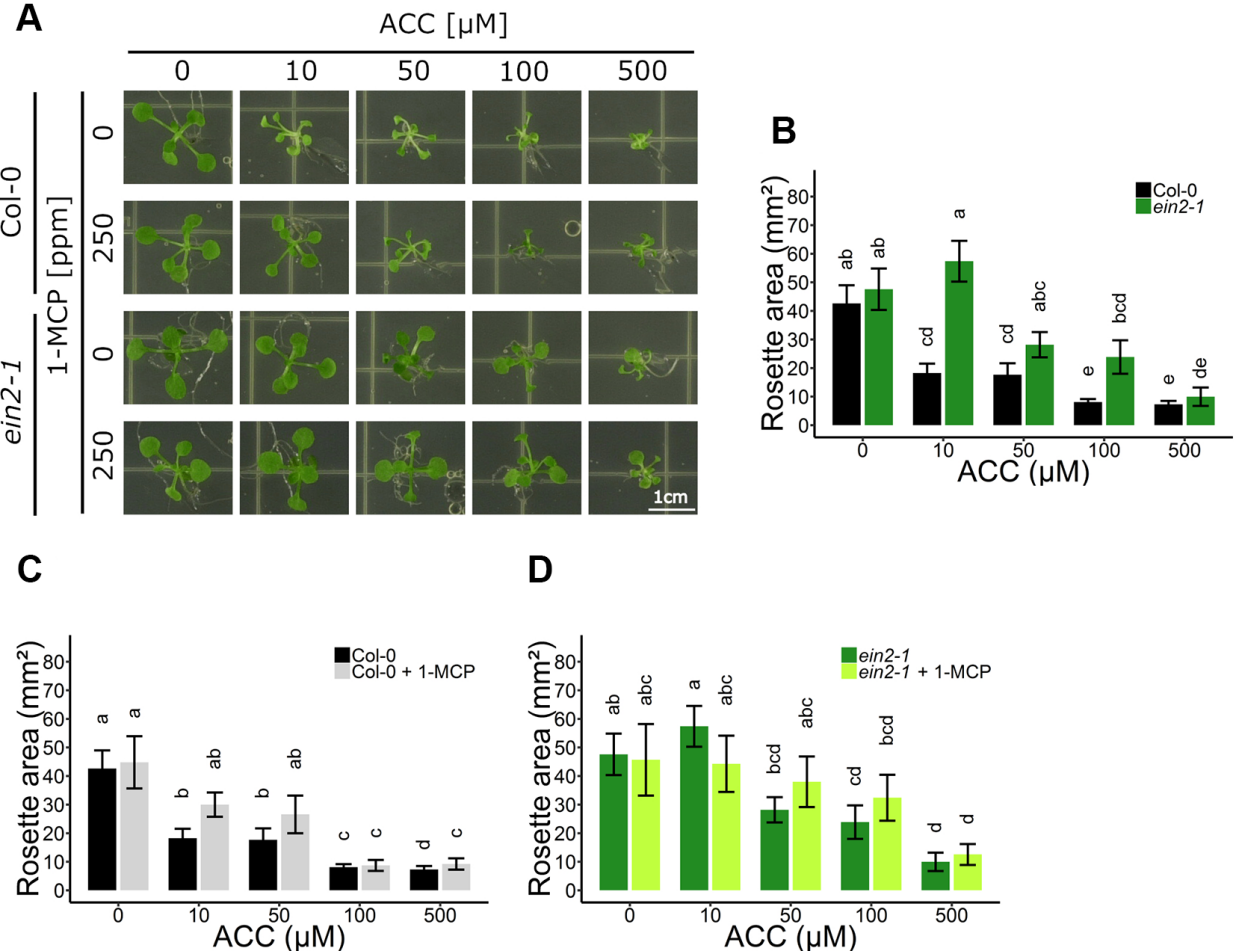
Rosette development of *Arabidopsis* wild-type Col-0 and ethylene insensitive *ein2-1* on increasing concentrations of ACC with and without 1-MCP treatment. *Arabidopsis* wild-type Col-0 and ethylene insensitive mutant *ein2-1* were sown on 0, 10, 50, 100, and 500 µM ACC with or without treatment with 250 ppm 1-MCP. **(A)** Pictures of representative 2-week-old plants under long-day conditions (16-h light/8-h dark) at light intensity of 70 μmol m^−2^ s^−1^ at 21°C. **(B)** Rosette area of 2-week-old Col-0 and *ein2-1* plants without 1-MCP treatment. **(C)** Rosette area of 2-week-old Col-0 plants. **(D)** Rosette area of 2-week-old *ein2-1* plants. Data from a representative experiment are shown; further corroborated by a minimum of three independent experiments. Different letters indicate statistically significant differences between the different groups (Kruskal-Wallis, Dunn’s Multiple Comparison Test, P < 0.01; 9 ≤ n ≤ 30). Error bars are SD. Effect sizes are presented in **Table S1**.

To explore the role of ACC in rosette development, possibly independent of ethylene, dose-response assays were conducted in 2-week-old WT Col-0 and in ethylene insensitive *ein2-1* plants (**Figures 1A, B**). In parallel, 1-methylcyclopropene (1-MCP) gas treatments, specifically blocking ethylene from binding to its receptor, were carried out to further investigate ACC-specific effects on rosette growth (**Figures 1C, D**). In WT Col-0, ACC reduced overall shoot growth in a dose-dependent manner, reflected by a decrease in rosette area (**Figures 1A, B**). Compared to the mock treatment, 10 µM ACC already reduced rosette area severely. A saturated response was visible as of 100 µM ACC. As expected, the phenotype of the rosettes was reminiscent of that of plants treated with exogenous ethylene or *ctr1* mutants, namely severe dwarfism caused by smaller leaf blades and petioles, as a result of inhibited cell expansion (Kieber et al., 1993; Rodrigues-Pousada et al., 1993). With a defective ethylene signaling pathway in *ein2-1*, rosettes responded differently to ACC compared to Col-0 (**Figures 1A, B**). At 10 µM ACC, *ein2-1* rosette size was slightly larger compared to a treatment with 0 µM ACC. Contrarily, at 100 µM ACC, the mean rosette area was decreased, as seen in Col-0 plants. The observation that ACC inhibited rosette development at higher doses regardless of the genotype, is indicative for an ethylene-independent effect of ACC. In addition, a small-scale experiment was carried out to determine whether the inhibitory effect of ACC on rosette growth is influenced by the presence of sucrose (**Figure S1**). In general, the omission of sucrose supplementation in the growth medium resulted in a decrease in rosette area in both Col-0 and *ein2-1* plants and irrespective of ACC concentration (**Figure S1B**). In the absence of ACC, a lack of sucrose resulted in a small inhibitory effect on rosette area in Col-0 and large decrease in *ein2-1*. However, at high concentrations of ACC (e.g., 100 µM) rosette area of Col-0 and *ein2-1* was reduced severely, irrespective of the presence of sucrose. However, the magnitude of the effect was larger upon sucrose supplementation. For instance, in *ein2-1*, 100 µM ACC decreased rosette area with 5.88 mm² and 12.30 mm² in the absence or presence of sucrose, respectively. Hence, although sucrose affects rosette growth in both WT and ethylene-insensitive plants, ACC is capable of inhibiting growth independently of ethylene and sucrose signaling.

When ethylene perception was blocked with 250 ppm 1-MCP, Col-0 rosettes were slightly larger compared to mock-treated rosettes, though this increase was negligible (**Figure 1C**). Furthermore, in the presence of 1-MCP, ACC was still able to reduce growth in a dose-dependent manner, indicating that ACC can affect shoot growth independently of ethylene perception. On 10 µM ACC, MCP-treated rosettes reached 30.01 mm² compared to 0 µM ACC, while 100 µM ACC further decreased rosette area to 8.72 mm² (**Figure 1C**). Nevertheless, in the presence of 1-MCP Col-0 exhibited a reduced sensitivity to ACC as compared to the absence of 1-MCP, consistent with the ACC dose-response in *ein2-1* (**Figures 1B, C**). Furthermore, 1-MCP did not substantially change the response of *ein2-1* to increasing concentrations of ACC (**Figures 1A, D**). An additional experiment using 50 ppm 1-MCP was conducted to verify that the observed inhibitory effects were not due to an excess of 1-MCP (**Figure S2**). The effects of ACC on rosette area were comparable to those in the presence of 250 ppm 1-MCP.

**Figure 2:**
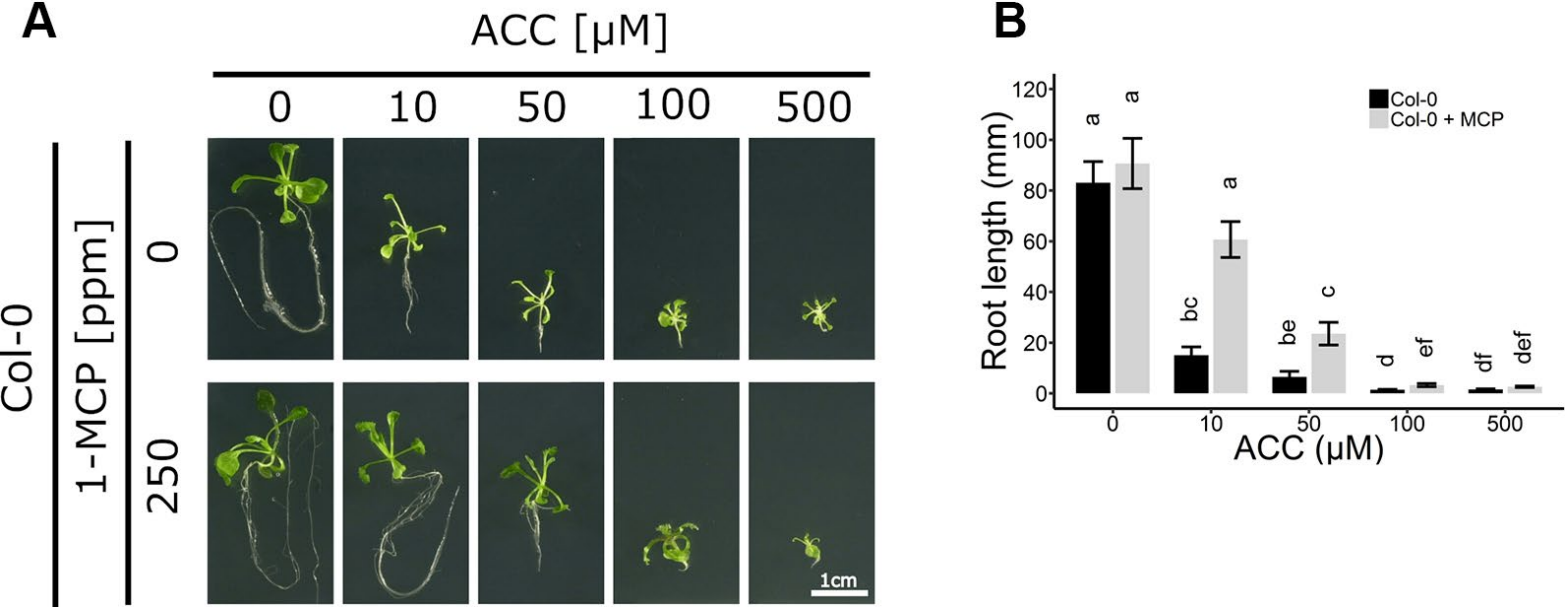
Root development of *Arabidopsis* wild-type Col-0 on increasing concentrations of ACC with and without 1-MCP treatment. *Arabidopsis* wild-type Col-0 was sown on 0, 10, 50, 100, and 500 µM ACC with or without treatment with 250 ppm 1-MCP. **(A)** Pictures of representative 2-week-old plants under long-day conditions (16-h light/8-h dark) at light intensity of 70 μmol m^−2^ s^−1^ at 21°C. **(B)** Root length of 2-week-old Col-0 plants. Data from a representative experiment are shown; further corroborated by a minimum of three independent experiments. Different letters indicate statistically significant differences between the different groups (Kruskal-Wallis, Dunn’s Multiple Comparison Test, P < 0.01; 28 ≤ n ≤ 44). Error bars are SD. Effect sizes are presented in **Table S1**.

ACC can also negatively affect root growth independently of ethylene signaling (**Figure 2A**). Col-0 seedlings were grown on increasing concentrations of ACC in the absence or presence of 250 ppm 1-MCP. Both ACC and 1-MCP dramatically altered root growth. In the absence of 1-MCP, a reduction in root length was already apparent at 10 µM ACC (**Figure 2B**), consistent with previously reported effects of ethylene on root growth inhibition (Le et al., 2001). In contrast, at the same concentration of ACC in the presence of 1-MCP, a much smaller inhibition was observed (**Figure 2B**). A five-fold higher dose was required for an effective inhibition of root elongation in Col-0 plants subjected to 1-MCP treatment (**Figures 2A, B**). It is conceivable that the observed effects of ACC on both rosette and root growth, in the presence of 1-MCP, are due to an ethylene signal remaining present under an insufficient dose of 1-MCP. However, the binding affinity of 1-MCP to the ethylene receptors was demonstrated to be at least 10 times greater compared to ethylene (Hall et al., 2000; Binder et al., 2004). Additionally, previous reports demonstrated that 500 ppb of ethylene is sufficient to mimic the phenotypic effects of 50 µM ACC at the level of rosette growth inhibition (Vaseva et al., 2018). Moreover, a treatment of etiolated seedlings with 1 mM ACC was shown to give rise to ethylene levels ranging from 1 to 10 ppm (Woeste et al., 1999). Altogether, these findings support the interpretation that 250 ppm of 1-MCP is more than sufficient to block the effects of 500 µM ACC. To further corroborate this assumption, plants were treated for 2 weeks with ethylene in the presence of 1-MCP to assess ethylene insensitivity (**Figure S3**). A dose of 100 ppm ethylene was chosen to effectively supersede the effects of 500 µM ACC. WT plants supplemented with ethylene displayed a typical dwarfed phenotype and reduced root growth, in the absence of 1-MCP (**Figure S3**). However, these phenotypic effects disappeared when ethylene perception was blocked with 100 ppm 1-MCP, or similarly in *ein2-1*, which has a defective ethylene signaling pathway and is fully unresponsive to ethylene. Thus, ACC, at the concentrations tested, is capable of reducing overall growth during early plant development, independently of ethylene.

### Effects of ACC on Vegetative Growth in the Presence of ACO Inhibitor AIB

**Figure 3:**
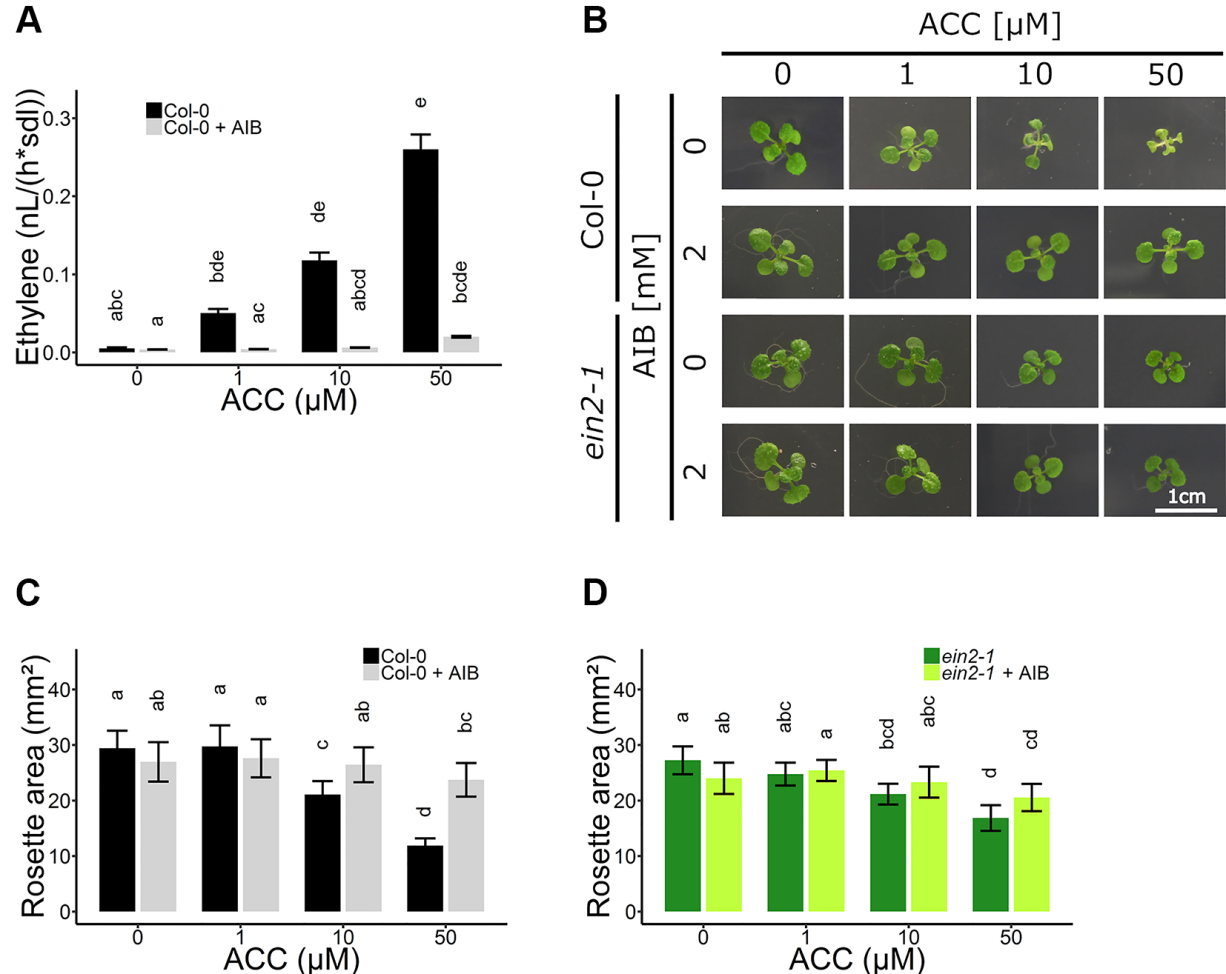
Rosette development of *Arabidopsis* wild-type Col-0 and ethylene insensitive *ein2-1* on increasing concentrations of ACC with and without AIB treatment. *Arabidopsis* wild-type Col-0 and ethylene insensitive mutant *ein2-1* were sown on 0, 10, and 50 µM ACC with or without treatment with 2 mM AIB. **(A)** Ethylene production rates of 2-week-old Col-0 plants (n = 3; 8 independent replicates). **(B)** Pictures of representative 2-week-old plants under long-day conditions (16-h light/8-h dark) at light intensity of 70 μmol m^−2^ s^−1^ at 21°C. **(C)** Rosette area of 2-week-old Col-0 plants (10 ≤ n ≤ 24; 3 independent replicates). **(D)** Rosette area of 2-week-old *ein2-1* plants (8 ≤ n ≤ 15; 3 independent replicates). Different letters indicate statistically significant differences between the different groups (Kruskal-Wallis, Dunn’s Multiple Comparison Test, P < 0.01). Error bars are SD. Effect sizes are presented in **Table S1**.

To further investigate the role of ACC in vegetative development, experiments using the ACO inhibitor AIB were conducted. Ethylene emanation was measured in 2-week-old plants grown on increasing doses of ACC in the absence and presence of 2 mM AIB (**Figure 3A**and **Table S2**). A dose-dependent increase in ethylene levels was observed when plants were grown on ACC-containing media. In addition, AIB effectively blocked ACO-mediated conversion of ACC to ethylene, though a small increase in ethylene levels, could be observed at higher concentrations (50 µM ACC; **Table S2**). Nevertheless, the ethylene levels in plants treated with 50 µM ACC + AIB were more than two-fold lower than those in plants treated with 1 µM ACC alone (**Figure 3A**and **Table S2**). Concentrations of ACC exceeding 50 µM were henceforth omitted, since in such conditions 2 mM AIB was unable to outcompete ACC for binding to the catalytic site of ACO, resulting in notable ethylene emanation. Moreover, if the phenotypic effect upon treatment with 50 µM ACC + AIB was stronger compared to that of 1 µM ACC alone, it was considered a *bona fide* ACC effect.

**Figure 4:**
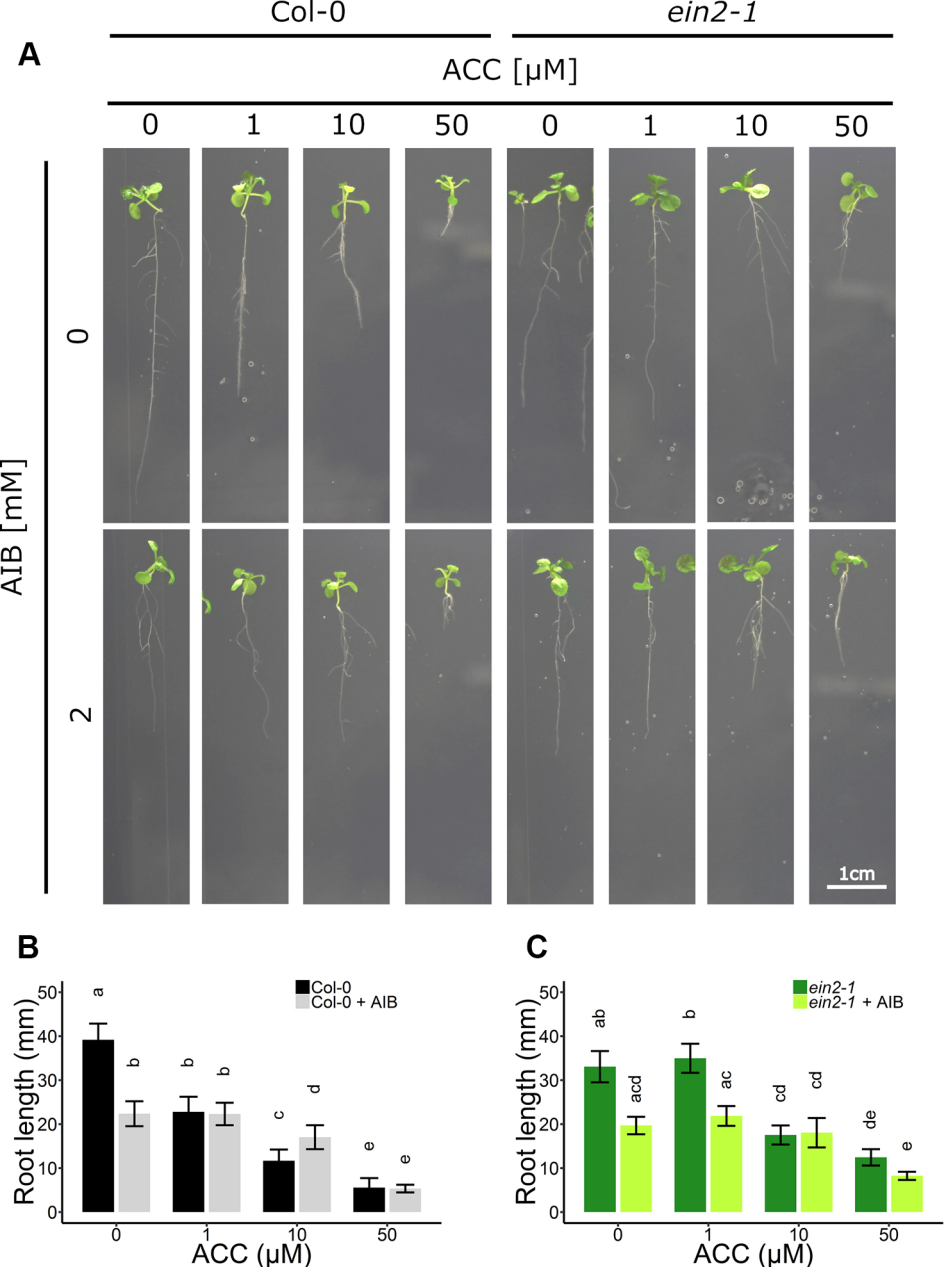
Root development of *Arabidopsis* wild-type Col-0 and ethylene insensitive *ein2-1* on increasing concentrations of ACC with and without AIB treatment. *Arabidopsis* wild-type Col-0 and ethylene insensitive mutant *ein2-1* were sown on 0, 1, 10, and 50 µM ACC with or without AIB treatment (2 mM). **(A)** Pictures of representative 2-week-old plants under long-day conditions (16-h light/8-h dark) at light intensity of 70 μmol m^−2^ s^−1^ at 21°C. **(B)** Root length of 2-week-old Col-0 plants (10 ≤ n ≤ 24; 3 independent replicates). **(C)** Root length of 2-week-old *ein2-1* plants (4 ≤ n ≤ 13; 3 independent replicates). Different letters indicate statistically significant differences between the different groups **(B)** One-way ANOVA, Tukey HSD; **(C)** Kruskal-Wallis, Dunn’s Multiple Comparison Test, P < 0.01). Error bars are SD. Effect sizes are presented in **Table S1**.

In subsequent experiments, the phenotypic responses upon AIB with increasing doses of ACC were investigated both at the level of rosette and root growth (**Figure 3B**). Fifty micromolar of ACC was capable of reducing rosette expansion of 2-week-old WT Col-0 plants treated with 1-MCP (**Figures 1A, B**). The addition of 2 mM AIB resulted in a slight decrease in rosette area upon 50 µM ACC, compared to 0 µM ACC (**Figures 3B, C**). In *ein2-1* plantlets, 50 µM ACC decreased rosette size substantially in the absence of AIB. However, in the presence of 2 mM AIB, rosette area reduced only slightly upon treatment with 50 µM ACC (**Figures 3B–D**). Contrarily, when roots were evaluated after 2 weeks of growth on vertically standing plates, a dose-dependent reduction in root length was discovered in Col-0 and *ein2-1* even in the presence of 2 mM AIB (**Figures 4A–C**). In Col-0, 50 µM ACC decreased the average root length from 39.17 mm to 5.57 mm in the absence of AIB. In the presence of AIB, root length was decreased from 22.37 mm to 5.35 mm (**Figure 4B**). Likewise, 50 µM ACC reduced root elongation substantially in *ein2-1* both without and with AIB supplementation (**Figure 4C**). Since the application of 50 µM ACC + AIB led to ethylene levels lower than those observed upon 1 µM ACC alone (**Figure 3A**), and the inhibitory effect of the latter dose was relatively small for both rosettes and roots, the stronger inhibitory effect observed on 50 µM ACC + AIB is assumed to be a true ACC effect (**Figures 3B, C** and **4B, C**). In conclusion, AIB hinders the growth-inhibiting effect of ACC in light-grown plantlets, in an organ-dependent manner.

### ACC Effects During Skotomorphogenic Development

**Figure 5:**
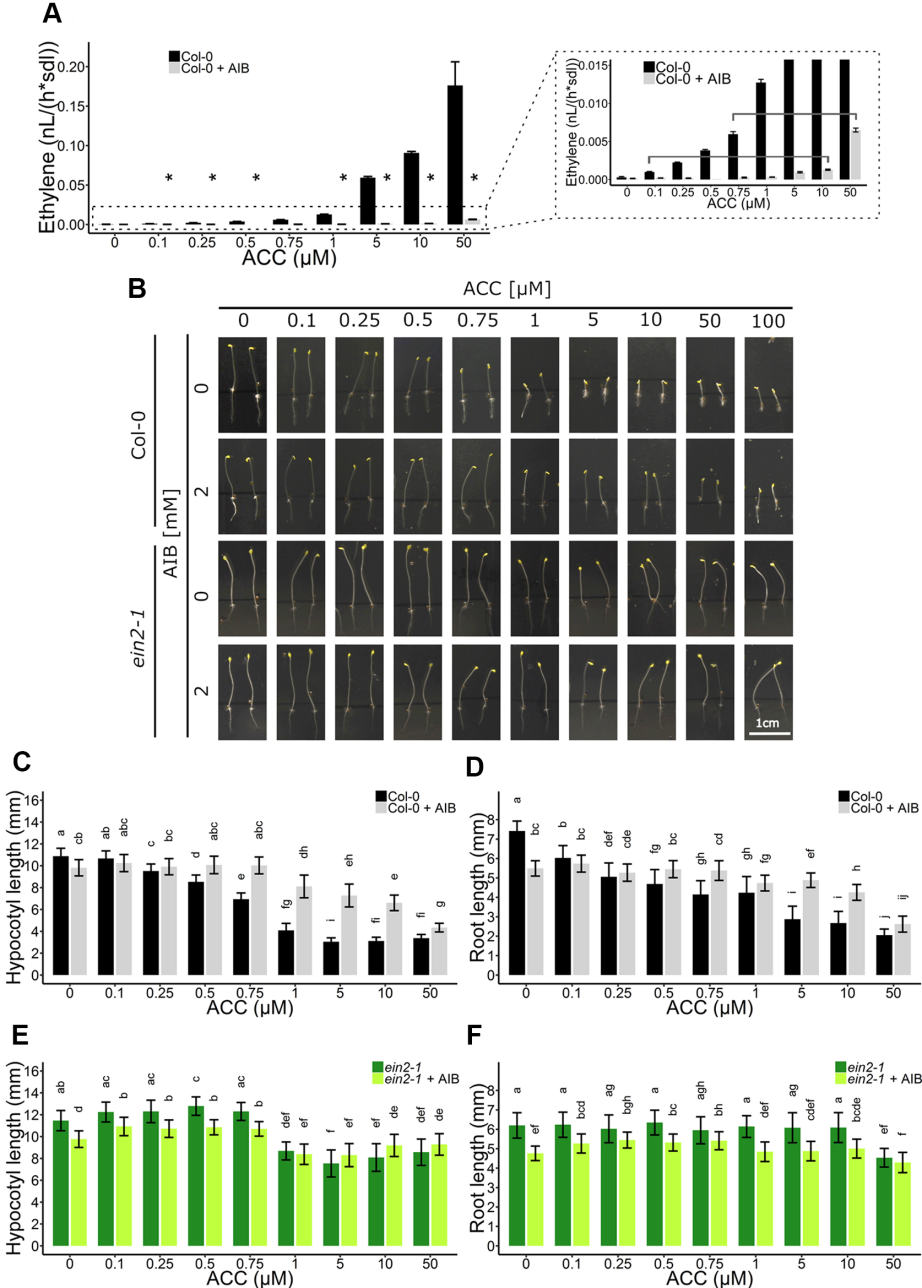
Triple response development of *Arabidopsis* wild-type Col-0 and ethylene insensitive *ein2-1* on increasing concentrations of ACC with and without AIB treatment. *Arabidopsis* wild-type Col-0 and ethylene insensitive mutant *ein2-1* were sown in darkness on 0, 0.1, 0.25, 0.5, 0.75, 1, 5, 10, and 50 µM ACC with or without treatment with 2 mM AIB. **(A)** Ethylene production rates of 4-day-old etiolated Col-0 seedlings (n = 30; 4 independent replicates). **(B)** Pictures of representative 4-day-old etiolated Col-0 and *ein2-1* seedlings. **(C)** Hypocotyl length and **(D)** root length of Col-0 seedlings (38 ≤ n ≤ 55; 3 independent replicates). **(E)** Hypocotyl length and **(F)** root length of *ein2-1* seedlings (22 ≤ n ≤ 42; 3 independent replicates). Different letters indicate statistically significant differences between the different groups (Kruskal-Wallis, Dunn’s Multiple Comparison Test, P < 0.01). Error bars are SD. The relative values of panels **(B**–**E)** are depicted in **Figure S4**. Effect sizes are presented in **Table S1**. P < 0.01 is indicated with *.

As ACC/ethylene affect skotomorphogenic development as well, an ethylene-independent role for ACC was investigated in etiolated seedlings (**Figure 5**). First, ethylene emanation by seedlings treated with ACC, AIB or a combination of both, was measured using laser-based photoacoustic spectroscopy (**Figure 5A**). AIB was able to effectively reduce ethylene synthesis to negligible levels up to doses of 10 µM ACC. At 10 µM ACC, AIB-treated seedlings emitted ethylene levels (µ = 1.29 pl seedling^−1^ h^−1^) comparable to seedlings subjected to 0.1 µM ACC without AIB (µ = 1.00 pl seedling^−1^ h^−1^) (**Table S3** and **Figure 5A**). A significant rise in ethylene levels could be observed at 50 µM ACC + AIB (µ = 6.50 pl seedling^−1^ h^−1^), which was similar to ethylene levels released upon treatment with 0.75 µM ACC alone (µ = 5.96 pl seedling^−1^ h^−1^) (**Table S3** and **Figure 5A**). Therefore, differences in phenotypic effects between the aforementioned treatments are indicative for true ACC effects.

Next, an AIB dose-response assay employing Col-0 and *ein2-1* was carried out on 4-day-old etiolated seedlings (**Figures 5B–F**). In Col-0, a dose-dependent inhibition of hypocotyl (**Figure 5C**; **Figure S4A**) and root (**Figures 5D** and **S4B**) elongation was observed, with roots being slightly more responsive to ACC at lower concentrations (as of 0.1 µM). Upon the addition of the ACO inhibitor AIB, hypocotyls demonstrated significant resistance to lower concentrations of ACC (0.5–10 µM), while reacting comparable to the wild type upon larger doses (>50 µM) (**Figure 5C**). For instance, 1 µM ACC reduced the average hypocotyl length from 10.88 mm to 4.08 mm, while in the presence of AIB, it only decreased from 9.81 mm to 8.11 mm (**Figure 5C**). In contrast, 50 µM ACC strongly inhibited hypocotyl length, irrespective of AIB treatment. A similar response was observed in AIB-treated roots (**Figure 5D**). In the ethylene-insensitive mutant *ein2-1*, ACC was able to significantly reduce hypocotyl and root growth as well, though to a much smaller extent than in Col-0 treated with AIB (**Figures 5B, E–F**and**S4C, D**). At 50 µM ACC and in the absence of AIB, hypocotyl length was merely reduced from 11.46 mm to 8.57 mm (**Figure 5E**).

Given that in Col-0, 10 and 50 µM ACC in the presence of AIB resulted in stronger inhibitory effects compared to 0.1 and 0.75 µM ACC, respectively, in the absence of AIB (**Figures 5B, C**), we hypothesized that these represent *bona fide* ACC responses. An ethylene complementation assay was conducted to rule out the possibility that residual ethylene biosynthesis upon AIB treatment is the cause of the observed phenotype in dark-grown seedlings (**Figure S5**). Specifically, the phenotypic effects of the residual levels of ethylene emanated by seedlings treated with either 10 µM ACC + AIB [designated ETH (10); 116 ppb] and 50 µM ACC + AIB [designated ETH (50); 585 ppb] were investigated (**Table S3**). When seedlings were treated with ETH (10), hypocotyls and roots were almost indistinguishable from the mock treatment (**Figures S5A–C**). In the presence of 2 mM AIB, the effect of ETH (10) was slightly larger in both organs. However, the effect of 10 µM ACC + AIB on hypocotyl and root elongation was stronger than that of ETH (10) [**Figures S5A–C**; compare ETH (10) with 10 µM ACC; gray bars]. Similarly, the effect of ETH (50) was less pronounced compared to 50 µM ACC + AIB [**Figures S5A–C**; compare ETH (50) with 50 µM ACC; gray bars]. Nevertheless, it is clear that the inhibitory effect on elongation of ETH (50) is stronger than that of ETH (10).

**Figure 6:**
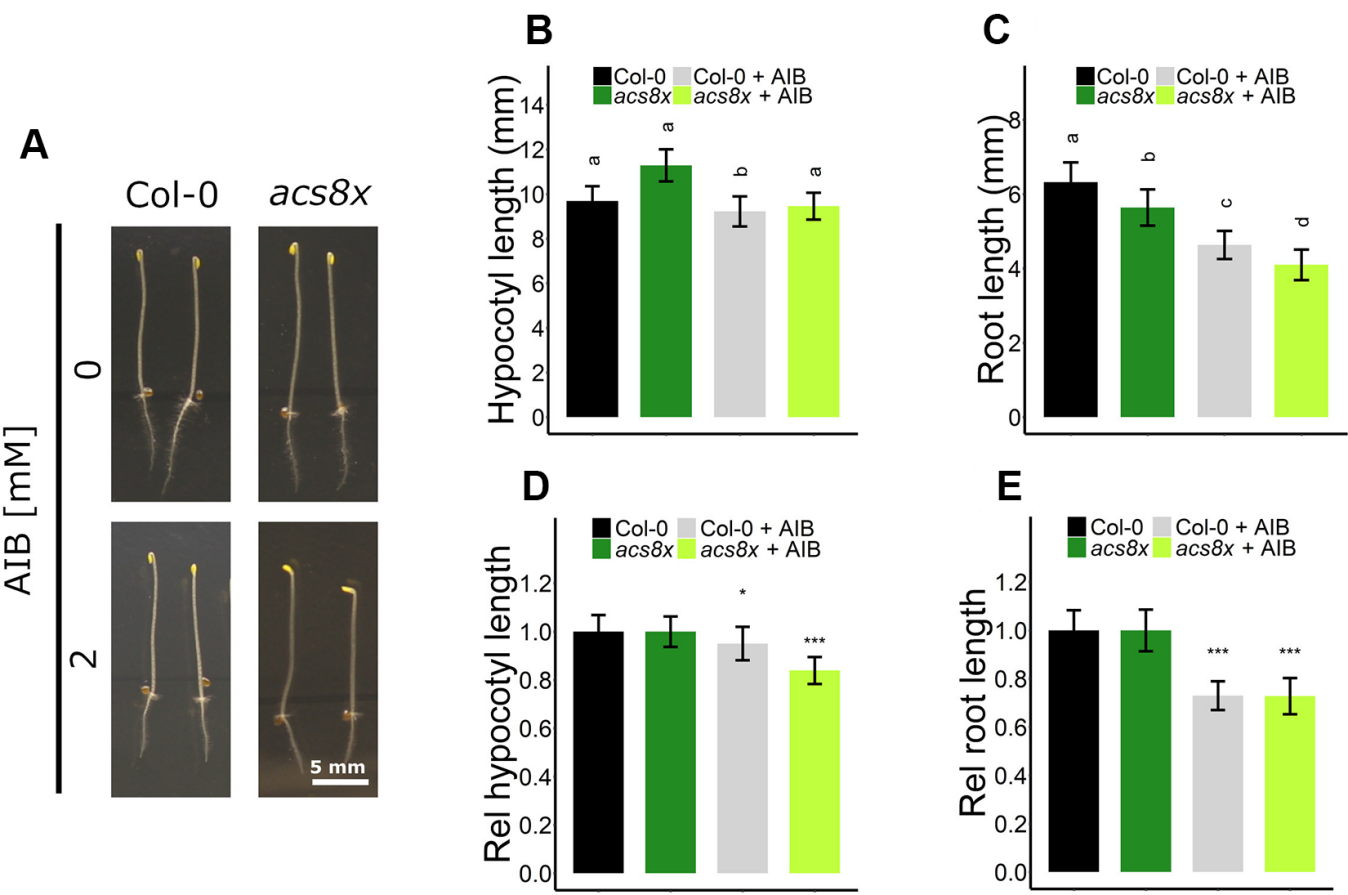
Triple response of *Arabidopsis* wild-type Col-0 and ethylene biosynthesis octuple mutant *acs8x* with and without AIB treatment. *Arabidopsis* wild-type Col-0 and ethylene biosynthesis octuple mutant acs8x were sown with or without AIB treatment (2 mM). **(A)** Pictures of representative 4-day-old etiolated Col-0 and *acs8x* seedlings. **(B)** Hypocotyl length and **(C)** root length of Col-0 and *acs8x* seedlings (13 ≤ n ≤ 26; 3 independent replicates). **(D)** Relative hypocotyl length and **(E)** relative root length of *acs8x* seedlings. In panels **(D)** and **(E)**, lengths are expressed relative to the corresponding genotype on medium without AIB. In panels **(B)** and **(C)**, different letters indicate statistically significant differences between the different groups (Kruskal-Wallis, Dunn’s Multiple Comparison Test, P < 0.01). T-tests were carried out in panels **(D)** and **(E)** to compare differences within a genotype (P < 0.01 is indicated with *; P < 0.0001 is indicated with ***). Error bars are SD. Effect sizes are presented in **Table S1**.

Interestingly, AIB reduced root length in both Col-0 and *ein2-1*, even in the absence of exogenous ACC or in the presence of ethylene (**Figures 4B, C**, **5D, F**, and **S5C**). In darkness, Col-0 and *ein2-1* roots were approximately 25% shorter when treated with 2 mM AIB (**Figures 5D, F**). To assess whether this inhibitory effect is caused by an accumulation of endogenous ACC or is due to a side effect of AIB, we investigated the response of the *acs8x* mutant, which is almost completely devoid of endogenous ACC, to AIB treatment (**Figure 6**). Etiolated *acs8x* seedlings exhibited significantly longer hypocotyls and shorter roots compared to the WT (**Figures 6A–C**), consistent with previous reports (Tsuchisaka et al., 2009). Upon addition of 2 mM AIB, both WT and *acs8x* roots were 30% shorter compared to roots in absence of AIB (**Figures 6C, E**). Furthermore, *acs8x* hypocotyls were slightly more sensitive to AIB compared to the WT (**Figures 6B, D**). These results strongly indicate that the response of seedlings to AIB is not related to an accumulation of ACC, as *acs8x* seedlings were not resistant to the treatment.

Altogether, these data corroborate an ethylene-independent role for ACC during skotomorphogenic shoot and root development (**Figure 5**), in addition to its negative effect on rosette and root development in light conditions (**Figures 1**and **4**). Furthermore, ACC evokes distinct responses in ethylene-sensitive compared to -insensitive backgrounds.

## Discussion

**Figure 7:**
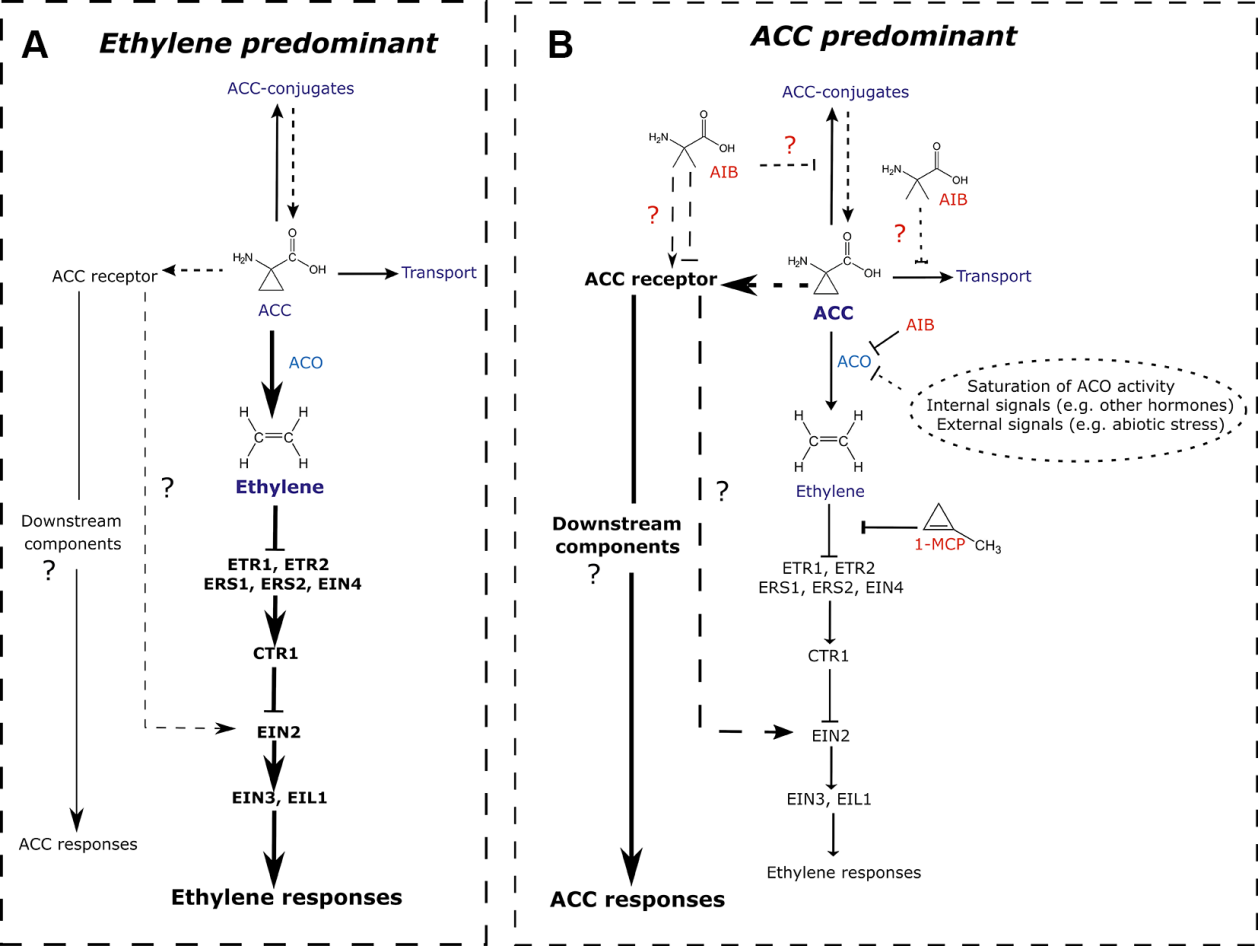
Revisited model for ACC/ethylene biosynthesis and signaling. **(A)** ACC/ethylene signaling pathway in normal conditions, where the responses are mainly mediated by ethylene. A major portion of ACC is converted to ethylene by ACO. The signal is then transferred *via* key signaling components ultimately leading to ethylene responses. The remaining ACC is transported to other tissues, conjugated to storage forms or perceived by (a) putative ACC receptor(s). Subsequent ACC responses are assumed to be minimal. **(B)** ACC/ethylene signaling pathway in conditions where ACC accumulates and leads to ACC-specific responses. ACC accumulation can result from an inhibition of ACO by inhibitors (e.g., AIB; shown in red), saturation of ACO activity (leading to overflow), or any other inhibition of ACO caused by internal or external signals. Elevated levels of ACC bind to its receptor(s), activating unidentified downstream components, ultimately leading to ACC-specific responses. Alternatively, ACC could, upon interaction with its receptor(s) act *via* EIN2, activating ethylene-mediated gene expression, while bypassing the need for ethylene perception. Additionally, the ACO inhibitor AIB potentially affects other ACC binding proteins such as putative receptors, transporters or conjugating enzymes. Arrow-headed lines represent stimulatory interactions. Bar-headed lines represent inhibitory interactions. Thick lines depict the predominantly active pathway. Dashed lines indicated relations that have not been demonstrated experimentally. 1-MCP, 1-methylcyclopropene; ACC, 1-aminocyclopropane-1-carboxylic acid; ACO, ACC oxidase; ACS, ACC synthase; AIB, 2-aminoisobutyric acid; CTR, constitutive triple response; ETR, ethylene response; ERS, ethylene response sensor; EIN, ethylene insensitive; EIL, EIN3-like.

Since the discovery of ACC as a biosynthetic precursor of the plant hormone ethylene about 4 decades ago (Adams and Yang, 1979), most studies were focused on identifying the mechanisms controlling ethylene synthesis and its subsequent *in vivo* responses. Few studies, however, investigated whether ACC has a non-canonical function, independent of ethylene signaling or bypassing ethylene perception. Here, we provide evidence for a role of ACC in the regulation of early vegetative development in *Arabidopsis thaliana.* Using chemical inhibitors of ethylene biosynthesis and perception, in WT and in the ethylene-insensitive mutant background *ein2-1*, our study demonstrates that ACC can act as a negative regulator of rosette development (**Figures 1**, **3** and **S1, S2**), hypocotyl elongation in darkness (**Figures 5** and **S4**), and root growth in both light (**Figures 2** and **4**) and etiolated conditions (**Figure 5**), in parallel of ethylene perception. Based on these findings, we propose to revisit the current model on ACC/ethylene biosynthesis and signaling, including ACC-specific responses (**Figure 7**).

In roots, ACC or ethylene treatment is known to cause growth inhibition through a reduction in LEH, in a concentration- and time-dependent manner (Le et al., 2001). Specifically, ethylene perception in the epidermal layer of the transition zone is sufficient to inhibit root growth (Vaseva et al., 2018). Moreover, this response is mediated by changes in intracellular auxin levels concomitant with an apoplastic alkalinization, which in turn regulates the activity of cell-wall loosening agents and peroxidases (Staal et al., 2011; Barbez et al., 2017; Vaseva et al., 2018). Though ACC and ethylene root responses are certainly overlapping, our data demonstrate that ACC can affect primary root growth independently of ethylene perception and/or signaling (**Figures 2**, **4**, and **5**). We propose that this role might be either specific to ACC or related to a feedback regulatory mechanism involving auxin, mimicking ethylene-mediated root inhibition (Vaseva et al., 2018). An ethylene-independent role for ACC as a signal driving root growth has been proposed previously (Xu et al., 2008; Tsang et al., 2011). They suggest that ACC is involved in the sensing of cell wall integrity, a feature crucial in the control of root elongation. It is conceivable that crosstalk between ACC and ethylene exists, since the latter also acts on factors related to cell wall integrity. Until now, no role for ACC in the regulation of hypocotyl or shoot growth has been thoroughly characterized. Higher order mutants of *ACS* genes, however, do exhibit reduced branching, a phenotype clearly distinct from single ethylene signaling mutants (Tsuchisaka et al., 2009). In addition, embryo lethality in a homozygous octuple *acs* mutant points to a primary role of ACC in early vegetative growth (Tsuchisaka et al., 2009). Contrarily, a non-canonical ethylene pathway, such as the controversial CTR1-MKK9–MPK3/MPK6-EIN3 signaling cascade, could regulate embryo development as well (Yoo et al., 2008). Analysis of an *ACO* null mutant or a heptuple *etr1ers1etr2ers2ein4ctr1ein2* will shed light on this issue. Our work supports a role for ACC as a regulator of hypocotyl and shoot growth, independently of ethylene (**Figures 1**, **3**, and **5C**). Ethylene is known to regulate hypocotyl elongation and leaf expansion *via* changes in cell wall integrity and microtubule orientation (Collett et al., 2000; Le et al., 2005; Pierik et al., 2007). In addition, downstream effects on auxin and gibberellin homeostasis are linked to ethylene-mediated shoot growth as well (Vriezen et al., 2004; Stepanova et al., 2007). It is unclear how ACC controls expansion in shoot tissues, but it could involve ACC-specific effects or depend on crosstalk with the abovementioned growth hormones too.

Sensitivity of plants to ACC or ethylene, as for other hormones (e.g., auxin) depends on the concentration, tissue, organ, developmental stage, species, and growth conditions (Abeles et al., 1992; Le et al., 2001; Vandenbussche et al., 2012; Vaseva et al., 2018). *Arabidopsis* roots are generally more sensitive to ACC/ethylene [> 0.1 µM in darkness (**Figure 5D**); > 1 µM in light (**Figure 4B**)] compared to hypocotyls (> 0.25 µM; **Figure 5C**) or rosettes (> 10 µM; **Figure 3C**). When ethylene effects were excluded using pharmacological or genetic approaches, a lower sensitivity to ACC could be observed in all tissues studied (**Figures 1**–**5**). The need for the application of relatively high doses of exogenous ACC is consistent with an "overflow model". Low doses of ACC are converted to ethylene by ACO (**Figure 7A**), whereas increasing concentrations of ACC are supposed to accumulate intracellularly due to ACO limitation, ultimately leading to ACC-specific responses (**Figure 7B**). Nevertheless, the possibility that phenotypic changes are due to toxicity rather than *bona fide* ACC effects when applied at higher doses cannot be excluded. However, this notion holds true for all pharmacological experiments with compounds that are not 100% pure. In addition, the activity of ACC most likely depends on the tissue, developmental stage, and environmental conditions. During flooding, for instance, ethylene evolution in tissues subjected to hypoxia is limited due to the lack of oxygen, a key factor for ACO activity (**Figure 7B**; Adams and Yang, 1979; Vanderstraeten and Van Der Straeten, 2017). On the one hand, ACC can act as a mobile signal, which is transported from root-to-shoot, where it can be converted to ethylene and induce the appropriate phenotypic response to stress (e.g., hyponasty, petiole and shoot elongation, etc.), if oxygen is present. On the other hand, upon complete submergence, ACC itself might act as a growth-limiting factor in roots and shoots, contributing to a quiescence strategy, eventually leading to enhanced survival. The precise role of ACC in these conditions should be further evaluated.

Whether ACC acts as a signal completely independent of ethylene signaling or whether it can interact with ethylene signaling components downstream of perception remains a key question to be resolved in future studies (**Figure 7B**). Given that a depletion of ACC confers embryo lethality and reduces branching, in contrast to ethylene insensitivity, distinct roles for ACC are probable (Tsuchisaka et al., 2009). Moreover, the stimulation of GMC division by ACC does not require the major ethylene signaling components, further hinting at an ethylene-independent ACC pathway (Yin et al., 2019). Tsang et al. (2011) have postulated the second option. ACC could act as a shortcut, bypassing the need for ethylene perception. In the latter scenario, ACC would have the capacity to elicit certain responses before the required threshold levels of ethylene are reached or when the synthesis of ethylene is hampered (e.g., upon hypoxia). Our results support the latter hypothesis, since—at least with respect to skotomorphogenic development—*ein2-1* is less sensitive to ACC compared to the WT supplemented with AIB (**Figures 5C–F**). Contrarily, a negative effect of ACC on rosette development or primary root development (in light conditions) appears to occur independently of ethylene (**Figures 1**–**4**), downstream or in parallel with EIN2. A thorough analysis employing different ethylene signaling mutants in combination with pharmacological treatments will shed light on the events occurring downstream of ACC. On the other hand, the differences in root growth inhibition by ACC could be stage- or light-dependent. For instance, a recent report demonstrated that root growth defects upon phosphate deficiency are related to blue light illumination, and are suppressed by growing roots in darkness, as usually the case in nature (Zhang et al., 2019). Furthermore, though we observe the same effect in the absence of sucrose, we cannot rule out the possibility that ACC effects are modulated by sucrose. Sucrose is known to affect various vegetative growth processes (Gibson, 2005). It was shown that 1% sucrose decreased hypocotyl length and enhanced root length in etiolated seedlings (Lu and Wen, 2019). Therefore, the degree of ethylene response diminished in hypocotyls, while being enhanced in roots. In addition, increasing concentrations of sugar reduces the stability of EIN3, the major transcription factor of the ethylene signaling pathway (Yanagisawa et al., 2003). Less is known about the effects of sucrose—and its interaction with ethylene—on rosette development. High levels of sucrose or glucose are inhibitory for growth (Zhou et al., 1995). Here, we demonstrated that 1% sucrose increased rosette size, though this effect disappeared upon increasing doses of ACC, irrespective of the genetic background (**Figure S1**). Hence, the growth inhibitory properties of ACC are not dependent on the presence of sucrose.

Interestingly, differences in ACC responsiveness could be observed between AIB and 1-MCP application. While hypocotyl or root growth was equally inhibited by ACC in the presence of AIB or 1-MCP (**Figures 2**, **4**, and **5**), rosettes were less responsive to ACC upon combination with AIB (**Figures 1** and **3**). These differences did not arise due to ethylene-related effects, as the concentrations used were considered sufficiently effective. Two millimolar of AIB effectively blocked ACO-mediated conversion of ACC to ethylene in plants treated with 50 µM ACC (**Figure 3B**). Furthermore, residual ethylene emanation was even lower compared to a treatment with 1 µM ACC without AIB (**Table S2**). For 1-MCP treatments, a dose of 100 ppm completely blocked the phenotypic effects induced by 100 ppm ethylene (**Figure S3A**). Therefore, notwithstanding the fact that a strict comparison remains difficult, the observed discrepancy is most likely caused by another factor. To further corroborate the results obtained with AIB, parallel analyses including higher order *aco* mutants as well as their crosses with *ein2-1* will be instrumental. Blocking the conversion of ACC to ethylene in a pentuple *aco* mutant would also lead to an excess of endogenous ACC similar to a treatment with AIB. However, such a mutant has not been constructed to date, and it remains to be seen whether it is even viable.

AIB was discovered as an ACO inhibitor, based on its structural similarities with ACC (Satoh and Esashi, 1982; Pirrung et al., 1998). Hence, it is plausible that AIB also has the capacity to interact with other ACC binding proteins, be it (a) putative ACC receptor(s), conjugating enzymes or ACC transporters (**Figure 7B**). The decrease in ACC sensitivity at the level of rosette expansion when AIB is present (**Figure 3**), hints at possible competition for binding to the putative ACC receptor. On the other hand, in the presence of AIB, one might expect an increase in endogenous ACC levels resulting from a feedback effect at the level of ACS, leading to ACC effects at lower concentrations of exogenous ACC. However, this is not supported by our analysis. Moreover, AIB still inhibited root growth in the *acs8x* mutant, suggesting that AIB exhibits side effects unrelated to the accumulation of ACC (**Figure 5**). No additive effects were observed upon a combined application of AIB and low concentrations of ACC (**Figure 5D**), indicative for the absence of a genuine side effect. Therefore, it is more conceivable that AIB and ACC act on the same target, such as a putative ACC receptor. Different ACC receptors could have different sensitivities to ACC or structural analogues (e.g., AIB) and can be expressed in a tissue-specific manner or are developmentally regulated, explaining the variation in AIB sensitivity among tissues and conditions. Furthermore, our data do not determine whether AIB exhibits agonist or antagonist properties for the putative ACC receptor(s). Lastly, it remains to be clarified whether ACC itself, a derivative or a downstream element is the *bona fide* signal. The function of all three ACC conjugates is largely unknown (Amrhein et al., 1981; Kionka and Amrhein, 1984; Martin et al., 1995). They are proposed to act as storage forms, depleting the pool of free ACC, but could also have signaling functions.

With this work, we demonstrated that ACC could function as a regulator of growth during early vegetative development, apart from its role as an ethylene precursor. Hence, researchers employing ACC as an ethylene precursor should be mindful of putative ACC effects confounding ethylene responses. The exact mechanism underlying the ACC response, however, remains to be identified. The discovery of a putative receptor will shed light on the molecular players involved in the ACC response, either directly or downstream. Additionally, phenotypic, genetic and transcriptomic analyses of higher order *aco* mutants and the identification of ACC transporters and conjugating enzymes *in vivo*, are merely few of the studies required to elucidate ACC metabolism and signaling. Though ACC research is still relatively uncharted territory, novel findings related to the non-canonical role of ACC and the related molecular mechanisms will open up exciting new avenues in plant hormone physiology, shedding light on the complex signaling networks shaping plant growth and development.

## Data Availability Statement

All datasets generated for this study are included in the article/**Supplementary Material**.

## Author Contributions

All authors conceived and designed the research. LV, TD, and SB conducted the experiments. LV, TD, and DS analyzed the data. LV, TD, and DS wrote the first draft of the manuscript. All authors read and commented on the manuscript. DS coordinated the project.

## Funding

DS gratefully acknowledges financial support from the Research Foundation Flanders (project G032717N) and Ghent University (Bijzonder Onderzoeksfonds, BOF-BAS 2018). LV is indebted to the Research Foundation Flanders for an SB-PhD fellowship (1S17917N).

## Conflict of Interest

The authors declare that the research was conducted in the absence of any commercial or financial relationships that could be construed as a potential conflict of interest.
