# Supplementary material for: The Ethylene Precursor ACC Affects Early Vegetative Development Independently of Ethylene Signaling
Source: Front Plant Sci. 2019 Dec 6;10:1591. doi: 10.3389/fpls.2019.01591 (PMC6908520; doi:10.3389/fpls.2019.01591)
Supplement: Supplementary file 1 [file DataSheet_1.pdf]

Supplementary Material

1 Supplementary Figures and Tables

1.1 Supplementary Figures

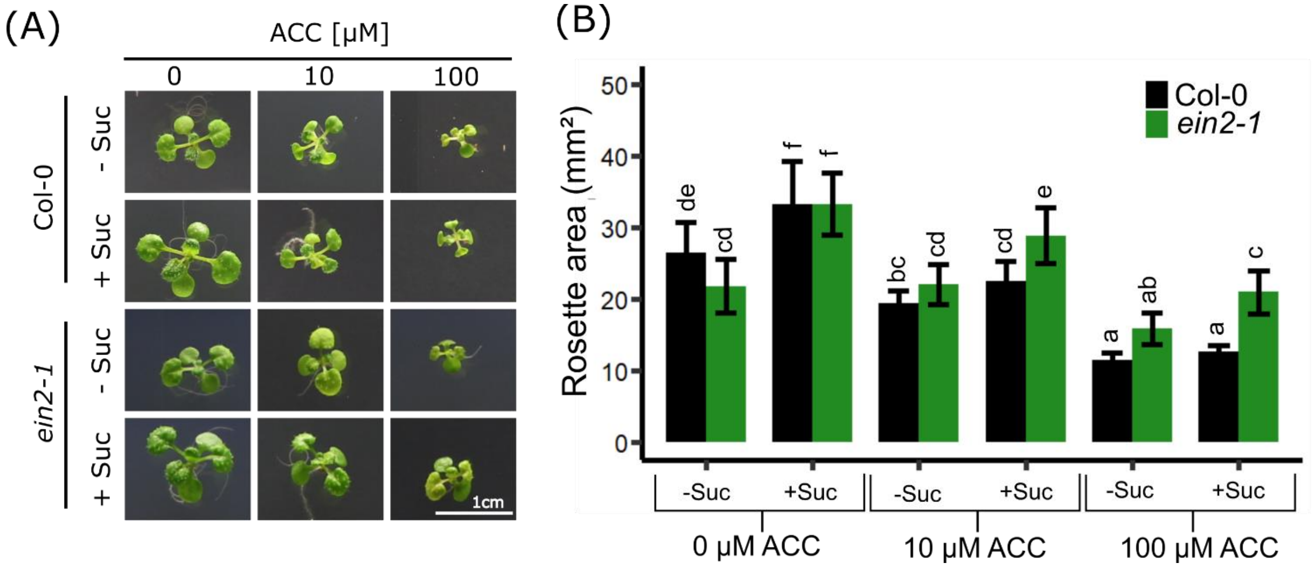

Supplementary Figure 1. Effect of sucrose supplementation on rosette development of *Arabidopsis* wild-type Col-0 and ethylene insensitive *ein2-1* on increasing concentrations of ACC. *Arabidopsis* wild-type Col-0 and ethylene insensitive mutant *ein2-1* were sown on 0, 10 and 100 µM ACC with 0 % (-Suc) or 1 % sucrose (+Suc). **(A)** Pictures of representative 2-week-old plants under long-day conditions (16h light/8h dark) at light intensity of 70 µmol m^{-2} s^{-1} at 21°C. **(B)** Rosette diameter of 2-week-old plants ($44 \leq n \leq 90$). Different letters indicate statistically significant differences between the different groups (Three-way ANOVA, Tukey HSD, $P < 0.01$). Error bars are SD. Effect sizes are presented in Table S1.

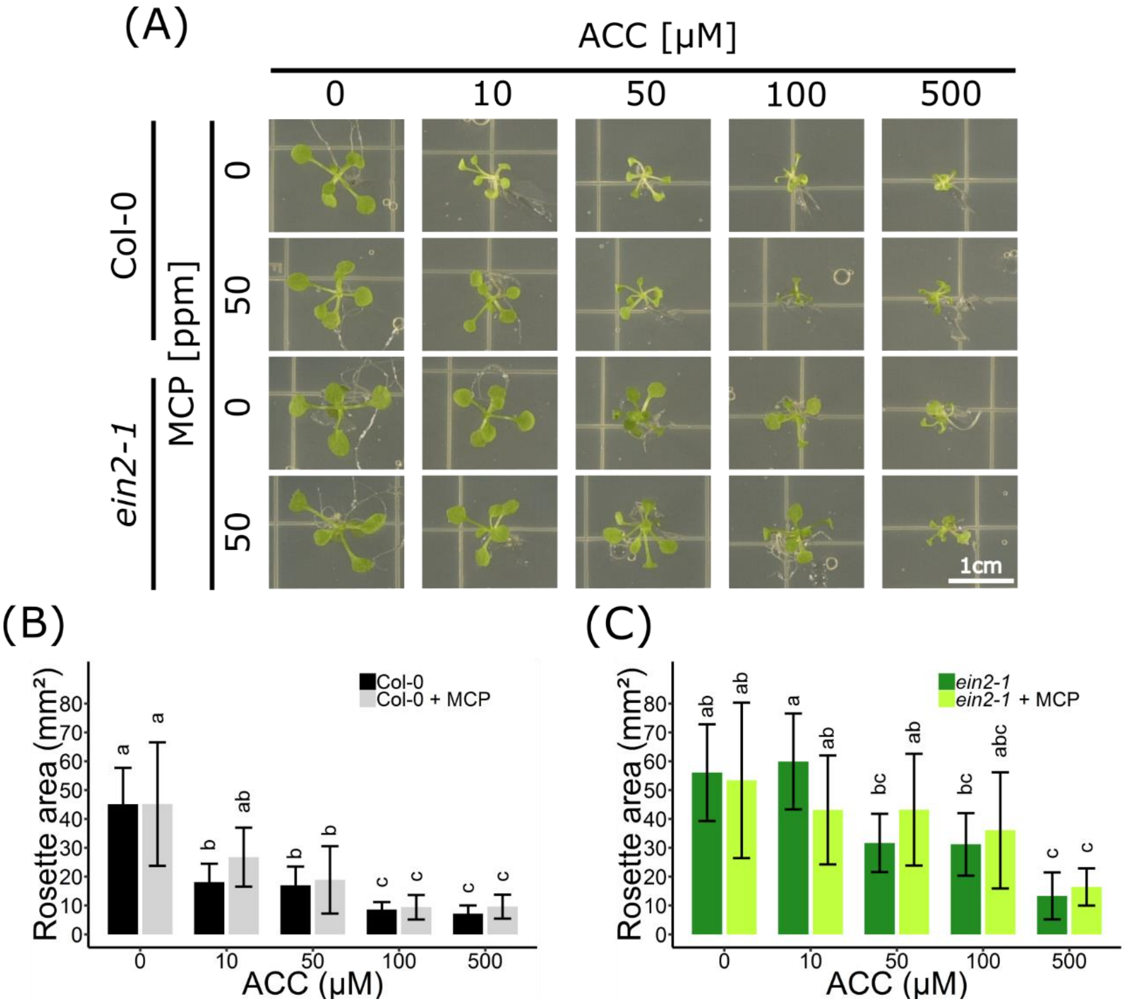

Supplementary Figure 2. Rosette development of *Arabidopsis* wildtype Col-0 and ethylene insensitive *ein2-1* on increasing concentrations of ACC with and without 1-MCP treatment. *Arabidopsis* wild-type Col-0 and ethylene insensitive mutant *ein2-1* were sown on 0, 10, 50, 100 and 500 µM ACC with or without treatment with 50 ppm 1-MCP. **(A)** Pictures of representative 2-week-old plants under long-day conditions (16h light/8h dark) at light intensity of 70 µmol m^{-2} s^{-1} at 21°C. **(B)** Rosette diameter of 2-week-old Col-0 plants ($21 \leq n \leq 29$). **(C)** Rosette diameter of 2-week-old *ein2-1* plants ($9 \leq n \leq 15$). Different letters indicate statistically significant differences between the different groups (Kruskal-Wallis, Dunn's Multiple Comparison Test, $P < 0.01$). Error bars are SD.

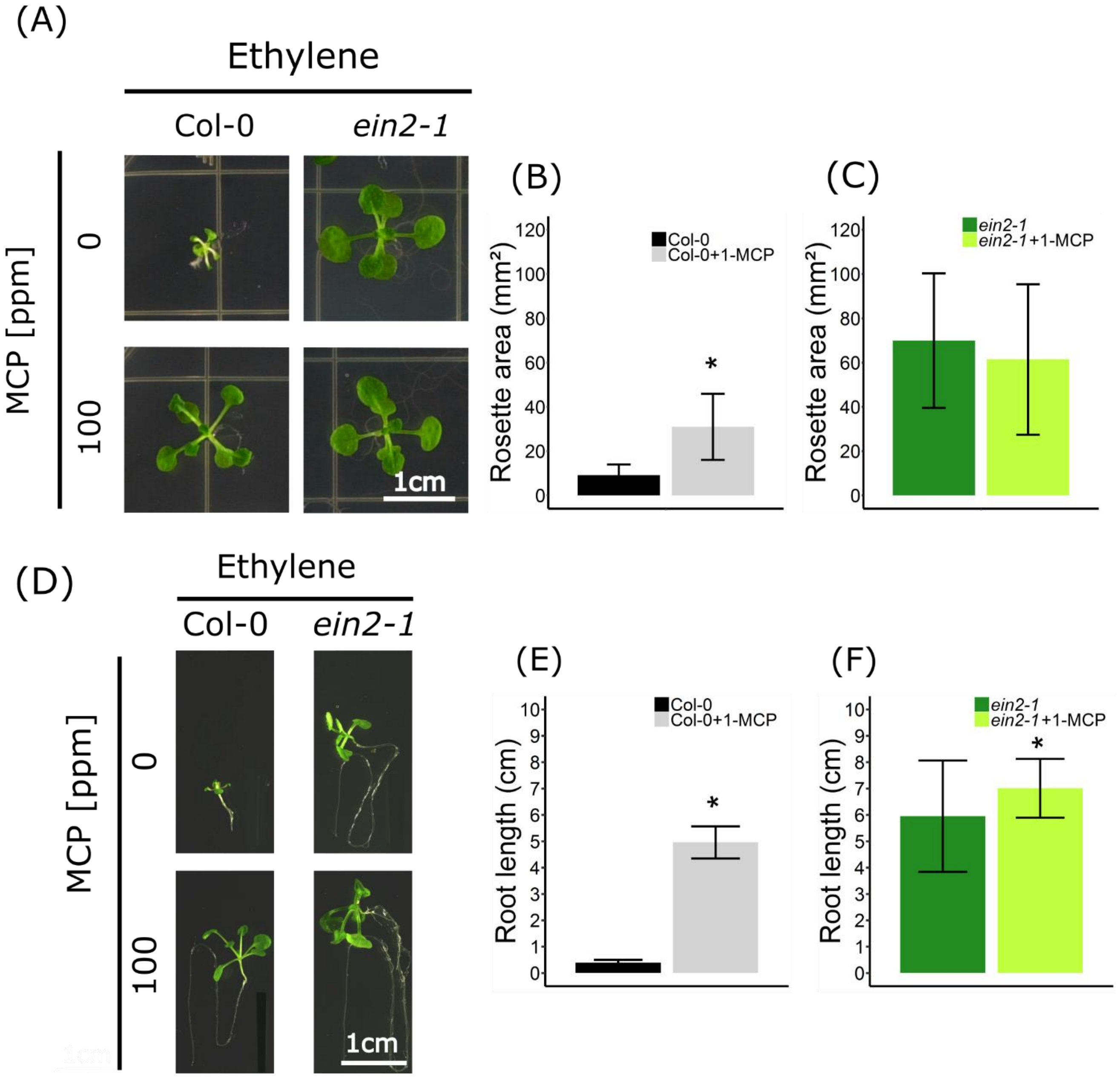

Supplementary Figure 3. Rosette and root development of *Arabidopsis* wild-type Col-0 and ethylene insensitive *ein2-1* under 100 ppm ethylene with and without 1-MCP treatment. *Arabidopsis* wild-type Col-0 and ethylene insensitive mutant *ein2-1* were grown in gassing chamber supplying 100 ppm ethylene with or without treatment with 100 ppm 1-MCP. **(A)** Pictures of representative horizontally grown 2-week-old plants under long-day conditions (16h light/8h dark) at light intensity of 70 µmol m^{-2} s^{-1} at 21°C. **(B)** Rosette diameter of 2-week-old Col-0 plants ($30 \leq n \leq 32$). **(C)** Rosette diameter of 2-week-old *ein2-1* plants ($27 \leq n \leq 29$). **(D)** Pictures of representative vertically grown 2-week-old plants under long-day conditions (16h light/8h dark) at light intensity of 70 µmol m^{-2} s^{-1} at 21°C. **(E)** Root length of 2-week-old Col-0 plants ($37 \leq n \leq 39$). **(F)** Root length of 2-week-old *ein2-1* plants ($29 \leq n \leq 36$). Statistical analysis was performed by use of a Mann-Whitney U Test (panels (B), (E) and (F), P <0.01) or a T-Test (Panel (C), P < 0.01). Error bars are SD.

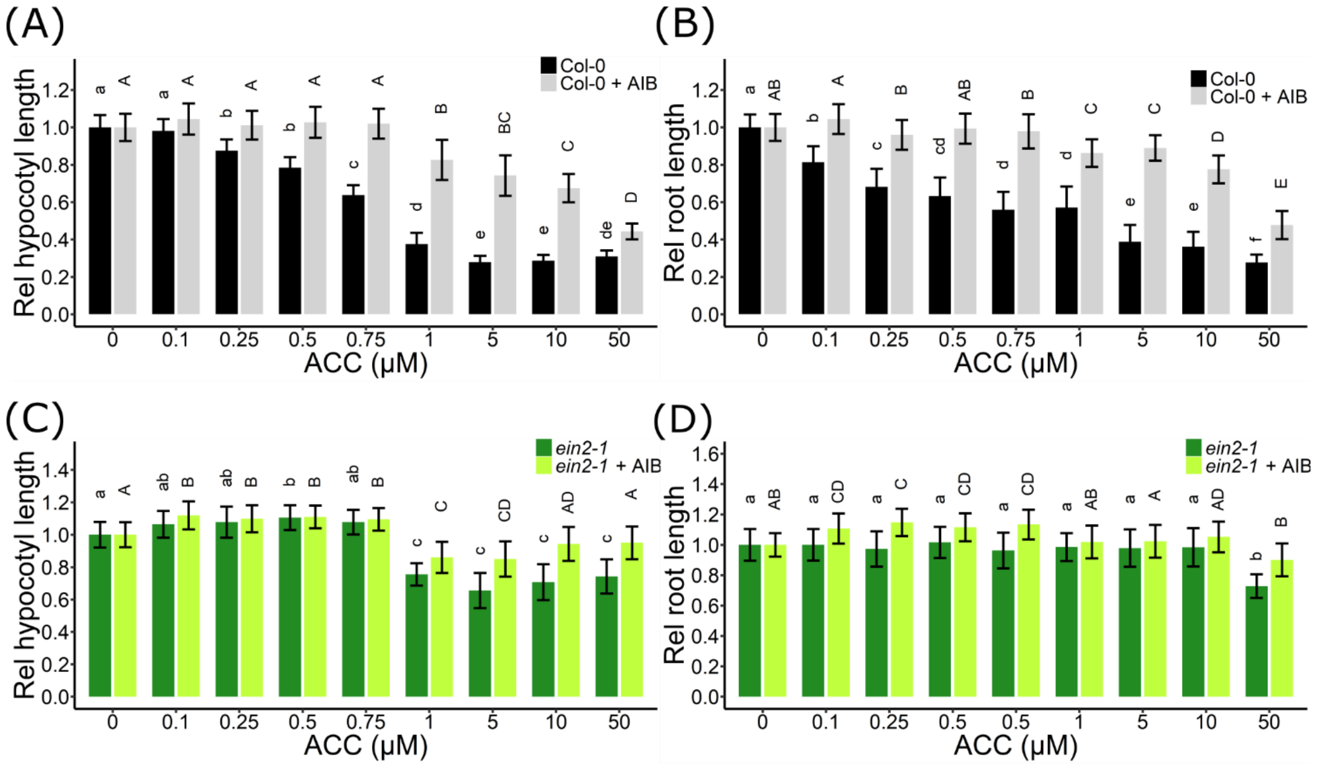

Supplementary Figure 4. Triple response of *Arabidopsis* wild-type Col-0 and ethylene insensitive *ein2-1* on increasing concentrations of ACC with and without AIB treatment. *Arabidopsis* wild-type Col-0 and ethylene insensitive mutant *ein2-1* were sown in darkness on 0, 0.1, 0.25, 0.5, 0.75, 1, 5, 10 and 50 µM ACC with or without treatment with 2 mM AIB. **(A)** Relative hypocotyl length and **(B)** relative root length of Col-0 seedlings ($38 \leq n \leq 55$; 3 independent replicates). **(C)** Relative hypocotyl length and **(D)** relative root length of *ein2-1* seedlings ($22 \leq n \leq 42$; 3 independent replicates). Lengths are expressed relative to the average length of seedlings treated with 0 µM ACC within each genotype and AIB concentration. Statistical analysis was conducted via Kruskal-Wallis and post hoc Dunn's Multiple Comparison Test ($P < 0.01$) for each genotype and AIB treatment. Different letters represent significant differences. Error bars are SD.

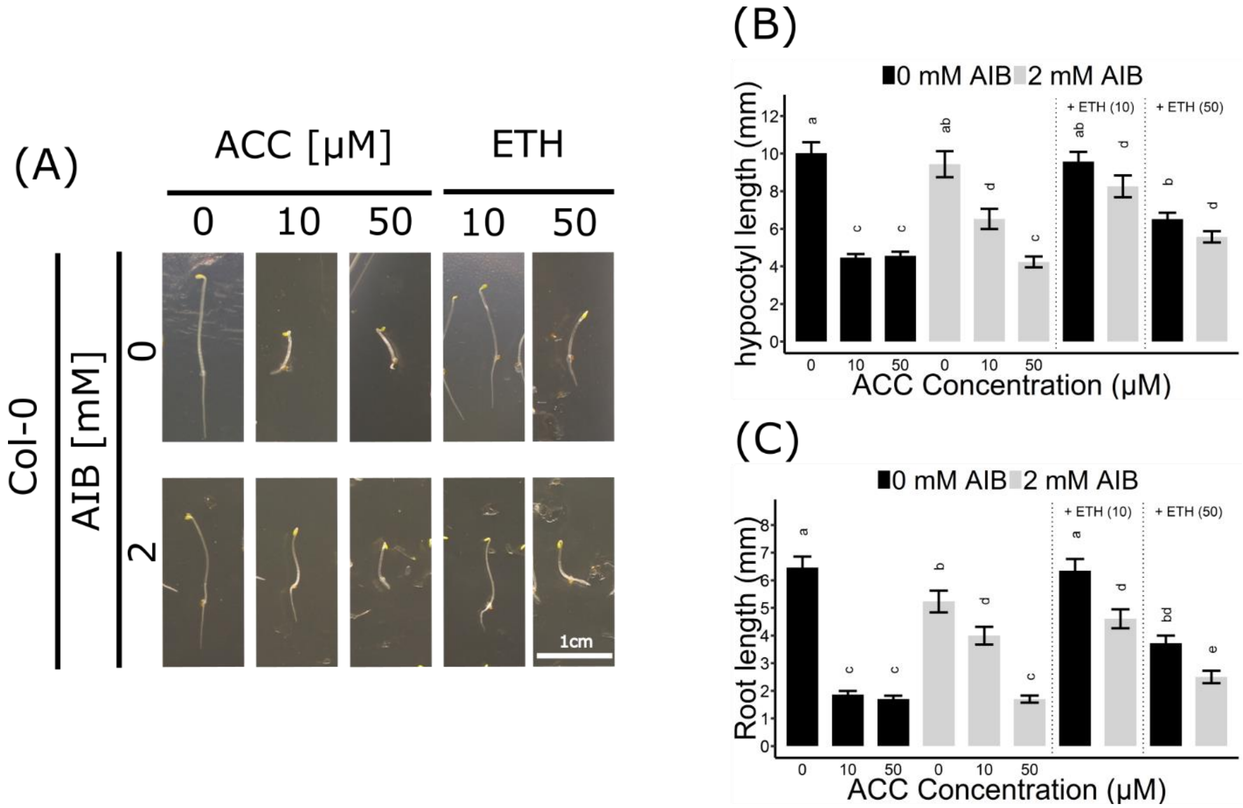

Supplementary Figure 5. Ethylene effects on etiolated *Arabidopsis* wild-type Col-0 seedlings in the presence with AIB treatment. Arabidopsis wild-type Col-0 were sown in darkness on 0, 10, and 50 µM ACC with or without treatment with 2 mM AIB. In addition wild-type Col-0 were sown in the absence of ACC with or without AIB treatment (2 mM), and supplemented at germination with residual ethylene levels ETH (10) (= 116 ppb) or ETH (50) (= 585 ppb). **(A)** Pictures of representative 4-day-old etiolated Col-0 seedlings. **(B)** Hypocotyl length and **(C)** root length of Col-0 seedlings (n = 30; 4 independent replicates). Different letters indicate statistically significant differences between the different groups (One-way ANOVA, Tukey HSD, $P < 0.01$). Error bars are SD. Effect sizes are presented in Table S1.

1.2. Supplementary Tables

Supplementary Table 1. Output of statistical analysis: effect sizes and p-values corresponding with statements made in the text. Small, medium and large effects correspond with effect sizes of 0.01, 0.06, and 0.15 for partial η² or 0.1, 0.3 and >0.5 for r.

Figure	Statement	Comparison		Effect size	P-value
Figure 1B	*Compared to the mock treatment, 10 µM ACC already reduced rosette area severely*	**Col-0 / 0 µM ACC**<br>Mean = 42.59 mm²<br>SD = 12.76 mm²	**Col-0 / 10 µM ACC**<br>Mean = 18.258 mm²<br>SD = 6.59 mm²	r = 0.56	*p* < 0.01
	A saturated response was visible as of 100 µM ACC	**Col-0 / 0 µM ACC**<br>Mean = 42.59 mm²<br>SD = 12.76 mm²	**Col-0 / 100 µM ACC**<br>Mean = 8.07 mm²<br>SD = 2.15 mm²	r = 1.06	*p* < 0.01
	At 10 µM ACC, ein2-1 rosette size was slightly larger compared to a treatment with 0 µM ACC.	***ein2-1* / 0 µM ACC**<br>Mean = 47.57 mm²<br>SD = 14.49 mm²	***ein2-1* / 10 µM ACC**<br>Mean = 57.39 mm²<br>SD = 14.28 mm²	r = 0.12	*p* = 0.58
	Contrarily, at 100 µM ACC, the mean rosette area was decreased	***ein2-1* / 0 µM ACC**<br>Mean = 47.57 mm²<br>SD = 14.49 mm²	***ein2-1* / 100 µM ACC**<br>Mean = 23.95 mm²<br>SD = 11.70 mm²	r = 0.49	*p* = 0.02
Figure S1B	*In general, the omission of sucrose supplementation in the growth medium resulted in a decrease in rosette area in both Col-0 and ein2-1 and irrespective of ACC concentration*	**Sucrose effect in three-way ANOVA analysis**		partial η² = 0.039	*p* < 0.01
	In the absence of ACC, a lack of sucrose resulted in a small inhibitory effect on rosette area in Col-0 and large decrease in ein2-1	**Col-0 / 0 µM ACC / +Suc**<br>Mean = 33.24 mm²<br>SD = 11.98 mm²	**Col-0 / 0 µM ACC / -Suc**<br>Mean = 26.43 mm²<br>SD = 8.71 mm²	r = 0.33	*p* < 0.01
		***ein2-1* / 0 µM ACC / +Suc**<br>Mean = 33.29 mm²<br>SD = 8.56 mm²	***ein2-1* / 0 µM ACC / -Suc**<br>Mean = 22.14 mm²<br>SD = 7.65 mm²	r = 0.62	*p* < 0.01
	However, at high concentrations of ACC (e.g. 100 µM) rosette area of Col-0 and ein2-1 was reduced severely, irrespective of the presence of sucrose	**ACC effect in three-way ANOVA analysis**		partial η² = 0.123	*p* < 0.01
	For instance, in ein2-1, 100 µM ACC decreased rosette area with 5.88 mm² and 12.30 mm² in the absence or presence of sucrose, respectively	***ein2-1* / 0 µM ACC / -Suc**<br>Mean = 22.14 mm²<br>SD = 7.65 mm²	***ein2-1* / 100 µM ACC / -Suc**<br>Mean = 15.93 mm²<br>SD = 4.43 mm²	r = 0.51	*p* < 0.01
		***ein2-1* / 0 µM ACC / +Suc**<br>Mean = 33.29 mm²<br>SD = 8.56 mm²	***ein2-1* / 100 µM ACC / +Suc**<br>Mean = 20.99 mm²<br>SD = 5.99 mm²	r = 0.66	*p* < 0.01

Figure 1C	*When ethylene perception was blocked with 250 ppm 1-MCP, Col-0 rosettes were slightly larger compared to mock-treated rosettes, though this increase was negligible*	**0 ppm 1-MCP / Col-0 / 0 µM ACC**<br>Mean = 42.59 mm^2<br>SD = 12.76 mm^2	**250 ppm 1-MCP / Col-0 / 0 µM ACC**<br>Mean = 44.78 mm^2<br>SD = 18.29 mm^2	r = 0.01	*p* = 0.93
	On 10 µM ACC, MCP-treated rosettes reached 30.01 mm² compared to 0 µM ACC, while 100 µM ACC further decreased rosette area to 8.72 mm²	**250 ppm 1-MCP / Col-0 / 0 µM ACC**<br>Mean = 44.78 mm^2<br>SD = 18.29 mm^2	**250 ppm 1-MCP / Col-0 / 10 µM ACC**<br>Mean = 30.01 mm^2<br>SD = 8.52 mm^2	r = 0.19	*p* = 0.25
		**250 ppm 1-MCP / Col-0 / 0 µM ACC**<br>Mean = 44.78 mm^2<br>SD = 18.29 mm^2	**250 ppm 1-MCP / Col-0 / 100 µM ACC**<br>Mean = 8.72 mm^2<br>SD = 3.80 mm^2	r = 1.04	*p* < 0.01
Figure 1D	*Furthermore, 1-MCP did not substantially change the response of ein2-1 to increasing concentrations of ACC*	**1-MCP effect in two-way ANOVA analysis**		partial $\eta^2 < 0.001$	*p* = 0.94
Figure 2B	*Both ACC and 1-MCP dramatically altered root growth*	**ACC effect in two-way ANOVA analysis**		partial $\eta^2 = 0.922$	*p* < 0.01
		**1-MCP effect in two-way ANOVA analysis**		partial $\eta^2 = 0.542$	*p* < 0.01
	In the absence of 1-MCP,a reduction in root length was already apparent at 10 µM ACC	**0 ppm 1-MCP / Col-0 / 0 µM ACC**<br>Mean = 83.07 mm<br>SD = 16.71 mm	**0 ppm 1-MCP / Col-0 / 10 µM ACC**<br>Mean = 15.02 mm<br>SD = 6.67 mm	r = 0.61	*p* < 0.01
	In contrast, at the same concentration of ACC in the presence of 1-MCP, a much smaller inhibition was observed (Fig. 2B; Table S1).	**250 ppm 1-MCP / Col-0 / 0 µM ACC**<br>Mean = 90.65 mm<br>SD = 19.83 mm	**250 ppm 1-MCP / Col-0 / 10 µM ACC**<br>Mean = 60.67 mm<br>SD = 14.22 mm	r = 0.24	*p* = 0.03
	A 5-fold higher dose was required for an effective inhibition of root elongation	**250 ppm 1-MCP / Col-0 / 0 µM ACC**<br>Mean = 90.65 mm<br>SD = 19.83 mm	**250 ppm 1-MCP / Col-0 / 50 µM ACC**<br>Mean = 23.53 mm<br>SD = 8.96 mm	r = 0.53	*p* < 0.01
Figure 3A	*A dose-dependent increase in ethylene levels was observed when plants were grown on ACC-containing media*	**ACC effect in two-way ANOVA analysis**		partial $\eta^2 = 0.889$	*p* < 0.01
	In addition, AIB effectively blocked ACO-mediated conversion of ACC to ethylene	**AIB effect in two-way ANOVA analysis**		partial $\eta^2 = 0.860$	*p* < 0.01
	though a small increase in ethylene levels, could be observed at higher concentrations	**0 µM ACC / 2 mM AIB**<br>Mean = 0.0038 pL seedling^{-1} h^{-1}<br>SD = 0.0003 pL seedling^{-1} h^{-1}	**50 µM ACC / 2 mM AIB**<br>Mean = 0.0200 pL seedling^{-1} h^{-1}<br>SD = 0.0023 pL seedling^{-1} h^{-1}	r = 0.75	*p* < 0.01
	Nevertheless, the ethylene levels in plants treated with 50 µM ACC + AIB were more than two-fold lower than those in plants treated with 1 µM ACC alone	**1 µM ACC / 0 mM AIB**<br>Mean = 0.0502 pL seedling^{-1} h^{-1}<br>SD = 0.0107 pL seedling^{-1} h^{-1}	**50 µM ACC / 2 mM AIB**<br>Mean = 0.0200 pL seedling^{-1} h^{-1}<br>SD = 0.0023 pL seedling^{-1} h^{-1}	r = 0.22	*p* = 0.44
Figure 3C	*The addition of 2 mM AIB resulted in a slight decrease*	**Col-0 / 0 µM ACC / 2 mM AIB**	**Col-0 / 50 µM ACC / 2 mM AIB**	r = 0.18	*p* = 0.11

	in rosette area upon 50 µM ACC	Mean = 26.96 mm^2<br>SD = 7.12 mm^2	Mean = 23.73 mm^2<br>SD = 6.03 mm^2		
	Since the application of 50 µM ACC + AIB led to ethylene levels lower than those observed upon 1 µM ACC alone (Fig. 3A), and the inhibitory effect of the latter dose was relatively small for both rosettes and roots	**Col-0 / 0 µM ACC / 0 mM AIB**<br>Mean = 29.40 mm^2<br>SD = 6.37 mm^2	**Col-0 / 1 µM ACC / 0 mM AIB**<br>Mean = 29.72 mm^2<br>SD = 7.12 mm^2	r = 0.21	*p* = 0.015
Figure 3D	*In ein2-1 plantlets, 50 µM ACC decreased rosette size substantially in the absence of AIB. However, in the presence of 2 mM AIB, rosette area reduced only slightly upon treatment with 50 µM ACC*	***ein2-1*** **/ 0 µM ACC / 0 mM AIB**<br>Mean = 27.25 mm^2<br>SD = 5.02 mm^2	***ein2-1*** **/ 50 µM ACC / 0 mM AIB**<br>Mean = 16.84 mm^2<br>SD = 4.64 mm^2	r = 0.73	*p* < 0.01
		ein2-1 **/ 0 µM ACC / 2 mM AIB**<br>Mean = 24.00 mm^2<br>SD = 5.66 mm^2	***ein2-1*** **/ 50 µM ACC / 2 mM AIB**<br>Mean = 20.54 mm^2<br>SD = 4.92 mm^2	r = 0.31	*p* = 0.03
Figure 4B	*In Col-0, 50 µM ACC decreased the average root length from 39.17 mm to 5.57 mm in the absence of AIB.*	**Col-0 / 0 µM ACC / 0 mM AIB**<br>Mean = 39.17 mm<br>SD = 7.37 mm	**Col-0 / 50 µM ACC / 0 mM AIB**<br>Mean = 5.57 mm<br>SD = 4.29 mm	r = 1.31	*p* < 0.01
	In the presence of AIB, root length was decreased from 22.37 mm to 5.35 mm	**Col-0 / 0 µM ACC / 2 mM AIB**<br>Mean = 22.37 mm<br>SD = 5.67 mm	**Col-0 / 50 µM ACC / 2 mM AIB**<br>Mean = 5.35 mm<br>SD = 1.77 mm	r = 0.86	*p* < 0.01
	Since the application of 50 µM ACC + AIB led to ethylene levels lower than those observed upon 1 µM ACC alone (Fig. 3A), and the inhibitory effect of the latter dose was relatively small for both rosettes and roots	**Col-0 / 0 µM ACC / 0 mM AIB**<br>Mean = 39.17 mm<br>SD = 7.37 mm	**Col-0 / 1 µM ACC / 0 mM AIB**<br>Mean = 22.78 mm<br>SD = 6.90 mm	r = 0.43	*p* < 0.01
Figure 4C	*Likewise, 50 µM ACC reduced root elongation substantially in ein2-1 both without and with AIB supplementation*	***ein2-1*** **/ 0 µM ACC / 0 mM AIB**<br>Mean = 33.06 mm<br>SD = 7.14 mm	***ein2-1*** **/ 50 µM ACC / 0 mM AIB**<br>Mean = 12.44 mm<br>SD = 3.74 mm	r = 0.94	*p* < 0.01
		ein2-1 **/ 0 µM ACC / 2 mM AIB**<br>Mean = 19.69 mm<br>SD = 3.97 mm	***ein2-1*** **/ 50 µM ACC / 2 mM AIB**<br>Mean = 8.25 mm<br>SD = 1.86 mm	r = 0.66	*p* < 0.01
Figure 5C	*In Col-0, a dose-dependent inhibition of hypocotyl elongation was observed*	**ACC effect in two-way ANOVA analysis**		partial η^2 = 0.729	*p* < 0.01
	For instance, 1 µM ACC reduced the average hypocotyl length from 10.88 mm to 4.08 mm, while in the presence of AIB, it only decreased from 9.81 mm to 8.11 mm	**Col-0 / 0 µM ACC / 0 mM AIB**<br>Mean = 10.88 mm<br>SD = 1.44 mm	**Col-0 / 1 µM ACC / 0 mM AIB**<br>Mean = 4.08 mm<br>SD = 1.29 mm	r = 1.08	*p* < 0.01
		**Col-0 / 0 µM ACC / 2 mM AIB**<br>Mean = 9.81 mm<br>SD = 1.48 mm	**Col-0 / 1 µM ACC / 2 mM AIB**<br>Mean = 8.11 mm<br>SD = 2.07 mm	r = 0.29	*p* < 0.01
	In contrast, 50 µM ACC strongly inhibited hypocotyl length, irrespective of AIB treatment	**Col-0 / 0 µM ACC / 0 mM AIB**<br>Mean = 10.88 mm<br>SD = 1.44 mm	**Col-0 / 50 µM ACC / 0 mM AIB**<br>Mean = 3.37 mm<br>SD = 0.70 mm	r = 1.19	*p* < 0.01
		**Col-0 / 0 µM ACC / 2 mM AIB**	**Col-0 / 50 µM ACC / 2 mM AIB**	r = 0.84	*p* < 0.01

		Mean = 9.81 mm<br>SD = 1.48 mm	Mean = 4.34 mm<br>SD = 0.80 mm		
	Given that in Col-0, 10 and 50 µM ACC in the presence of AIB resulted in stronger inhibitory effects compared to 0.1 and 0.75 µM ACC, respectively, in the absence of AIB	**Col-0 / 0 µM ACC / 2 mM AIB**<br>Mean = 9.81 mm<br>SD = 1.48 mm	**Col-0 / 10 µM ACC / 2 mM AIB**<br>Mean = 4.34 mm<br>SD = 0.80 mm	r = 0.54	$p < 0.01$
		**Col-0 / 0 µM ACC / 0 mM AIB**<br>Mean = 10.88 mm<br>SD = 1.44 mm	**Col-0 / 0.1 µM ACC / 0 mM AIB**<br>Mean = 10.66 mm<br>SD = 1.40 mm	r = 0.03	$p = 0.61$
		**Col-0 / 0 µM ACC / 0 mM AIB**<br>Mean = 10.88 mm<br>SD = 1.44 mm	**Col-0 / 0.75 µM ACC / 0 mM AIB**<br>Mean = 6.93 mm<br>SD = 1.15 mm	r = 0.70	$p < 0.01$
Figure 5D	*In Col-0, a dose-dependent inhibition of root elongation was observed*	**ACC effect in two-way ANOVA analysis**		partial η^2 = 0.523	$p < 0.01$
	Given that in Col-0, 10 and 50 µM ACC in the presence of AIB resulted in stronger inhibitory effects compared to 0.1 and 0.75 µM ACC, respectively, in the absence of AIB	**Col-0 / 0 µM ACC / 2 mM AIB**<br>Mean = 5.49 mm<br>SD = 0.79 mm	**Col-0 / 10 µM ACC / 2 mM AIB**<br>Mean = 4.26 mm<br>SD = 0.82 mm	r = 0.44	$p < 0.01$
		**Col-0 / 0 µM ACC / 2 mM AIB**<br>Mean = 5.49 mm<br>SD = 0.79 mm	**Col-0 / 50 µM ACC / 2 mM AIB**<br>Mean = 2.62 mm<br>SD = 0.82 mm	r = 1.11	$p < 0.01$
		**Col-0 / 0 µM ACC / 0 mM AIB**<br>Mean = 7.41 mm<br>SD = 1.02 mm	**Col-0 / 0.1 µM ACC / 0 mM AIB**<br>Mean = 5.74 mm<br>SD = 0.87 mm	r = 0.30	$p < 0.01$
		**Col-0 / 0 µM ACC / 0 mM AIB**<br>Mean = 7.41 mm<br>SD = 1.02 mm	**Col-0 / 0.75 µM ACC / 0 mM AIB**<br>Mean = 5.38 mm<br>SD = 1.02	r = 0.86	$p < 0.01$
	In darkness, Col-0 and ein2-1 roots were approximately 25% shorter when treated with 2 mM AIB	**Col-0 / 0 µM ACC / 0 mM AIB**<br>Mean = 7.41 mm<br>SD = 1.02 mm	**Col-0 / 0 µM ACC / 2 mM AIB**<br>Mean = 5.49 mm<br>SD = 0.79 mm	r = 0.42	$p < 0.01$
Figure 5E	*At 50 µM ACC and in the absence of AIB, hypocotyl length was merely reduced from 11.46 mm to 8.57 mm*	***ein2-1*** **/ 0 µM ACC / 0 mM AIB**<br>Mean = 11.46 mm<br>SD = 1.85 mm	***ein2-1*** **/ 50 µM ACC / 0 mM AIB**<br>Mean = 8.57 mm<br>SD = 2.41 mm	r = 0.58	$p < 0.01$
Figure 5F	*In darkness, Col-0 and ein2-1 roots were approximately 25% shorter when treated with 2 mM AIB*	***ein2-1*** **/ 0 µM ACC / 0 mM AIB**<br>Mean = 6.19 mm<br>SD = 1.31 mm	***ein2-1*** **/ 0 µM ACC / 2 mM AIB**<br>Mean = 4.76 mm<br>SD = 0.74 mm	r = 0.59	$p < 0.01$
Figure 5SB	*When seedlings were treated with ETH (10), hypocotyls and roots were almost indistinguishable from the mock treatment*	**0 µM ACC / 0 mM AIB**<br>Mean = 10.03 mm<br>SD = 1.15 mm	**ETH (10) / 0 mM AIB**<br>Mean = 9.57 mm<br>SD = 1.03 mm	r = 0.06	$p = 0.35$
	In the presence of 2 mM AIB, the effect of ETH (10) was slightly larger in both organs	**0 µM ACC / 2 mM AIB**<br>Mean = 9.43 mm<br>SD = 1.38 mm	**ETH (10) / 2 mM AIB**<br>Mean = 8.25 mm<br>SD = 1.15 mm	r = 0.17	$p = 0.04$
	However, the effect of 10 µM ACC + AIB on hypocotyl and	**0 µM ACC / 2 mM AIB**<br>Mean = 9.43 mm	**10 µM ACC / 2 mM AIB**<br>Mean = 6.52 mm	r = 0.51	$p < 0.01$

	root elongation was stronger than that of ETH (10)	SD = 1.38 mm	SD = 1.06 mm		
Figure 6B	*Etiolated acs8x seedlings exhibited significantly longer hypocotyls and shorter roots compared to the wild-type*	**Col-0 / 0 mM AIB**<br>Mean = 9.69 mm<br>SD = 1.33 mm	***acs8x* / 0 mM AIB**<br>Mean = 11.29 mm<br>SD = 0.79 mm	r = 0.58	*p* < 0.01
Figure 6C	*Etiolated acs8x seedlings exhibited significantly longer hypocotyls and shorter roots compared to the wild-type*	**Col-0 / 0 mM AIB**<br>Mean = 6.32 mm<br>SD = 1.06 mm	***acs8x* / 0 mM AIB**<br>Mean = 5.64 mm<br>SD = 0.98 mm	r = 0.46	*p* < 0.01
	Upon addition of 2 mM AIB, both wild-type and acs8x roots were 30 % shorter compared to roots in absence of AIB (Fig. 6C, E)	**Col-0 / 0 mM AIB**<br>Mean = 6.32 mm<br>SD = 1.06 mm	**Col-0 / 2 mM AIB**<br>Mean = 4.63 mm<br>SD = 0.75 mm	r = 0.67	*p* < 0.01
		**acs8x / 0 mM AIB**<br>Mean = 5.64 mm<br>SD = 0.98 mm	**acs8x / 2 mM AIB**<br>Mean = 4.10 mm<br>SD = 0.82 mm	r = 0.67	*p* < 0.01

Supplementary Table 2. Average ethylene emanation (SD) of 2-week-old light grown *Arabidopsis* wild-type Col-0 seedlings treated with ACC, AIB or a combination of both.

Treatment ACC	Treatment 0 mM AIB (in pL/h/plant)	Treatment 2 mM AIB (in pL/h/plant)
**0 μM**	5.25 (2.42)	3.73 (0.31)
**1 μM**	50.26 (10.76)	4.17 (0.54)
**10 μM**	117.78 (20.15)	6.19 (0.62)
**50 μM**	259.92 (38.41)	20.07 (2.31)

Supplementary Table 3. Average ethylene emanation (SD) of etiolated 4-day-old *Arabidopsis* wild-type Col-0 seedlings treated with ACC, AIB or a combination of both.

Treatment ACC	Treatment 0 mM AIB (in pL/h/seedling)	Treatment 2 mM AIB (in pL/h/seedling)
**0 μM**	0.29 (0.21)	0.14 (0.08)
**0.1 μM**	1.00 (0.13)	0.20 (0.06)
**0.25 μM**	2.23 (0.14)	0.18 (0.09)
**0.5 μM**	2.87 (1.93)	0.06 (0.01)
**0.75 μM**	5.96 (0.67)	0.27 (0.13)
**1 μM**	12.73 (0.86)	0.32 (0.08)
**5 μM**	59.29 (3.28)	0.97 (0.18)
**10 μM**	90.75 (3.71)	1.29 (0.16)
**50 μM**	176.18 (59.87)	6.50 (0.52)
